# PcWRKY1 Represses Transcription of *Yellow Stripe‐Like 3* (*PcYSL3*) to Negatively Regulate Radial Cadmium Transport in Poplar Stems

**DOI:** 10.1002/advs.202405492

**Published:** 2024-11-11

**Authors:** Xin Chen, Yuhong Zhang, Yao Cheng, Wenjian Yu, Lingyu Yang, Peiqi Shu, Jing Zhou, Payam Fayyaz, Zhi‐Bin Luo, Shurong Deng, Wenguang Shi

**Affiliations:** ^1^ State Key Laboratory of Tree Genetics and Breeding Research Institute of Forestry Chinese Academy of Forestry Beijing 100091 P. R. China; ^2^ Forest, Range and Watershed Management Department Agriculture and Natural Resources Faculty Yasouj University Yasuj 75919 63179 Iran; ^3^ Institute of Ecological Conservation and Restoration Chinese Academy of Forestry Beijing 100091 P. R. China; ^4^ Comprehensive Experimental Center of Chinese Academy of Forestry in Yellow River Delta Dongying Shandong 257000 P. R. China

**Keywords:** heavy metal, populus, transcriptional regulation, transporter, xylem‐to‐phloem transport

## Abstract

A considerable amount of cadmium (Cd) can accumulate in the bark of poplar stems, but the Cd transport pathway and its underlying molecular mechanisms remain unknown. Here, a Cd radial transport pathway in poplar stems and a previously unrecognized PcWRKY1‐*Yellow Stripe‐Like 3* (*PcYSL3*) module that regulates Cd transport are identified in *Populus* × *canescens* (Aiton) Sm. Cadmiun‐nicotianamine (Cd‐NA) in xylem vessels in poplar stem‐wood is unloaded to adjacent ray parenchyma cells and further radially transported to bark‐phloem. *PcYSL3* is putatively identified as involved in Cd radial transport in poplar stems. *PcYSL3* is highly expressed in ray parenchyma cells adjacent to xylem vessels and the encoded protein localizes on the plasma membrane. Cd accumulation is greater in the wood and bark of *PcYSL3*‐overexpressing poplars than the wild type, whereas the opposite is observed in *PcYSL3*‐knockdown plants. PcWRKY1 can bind to the *PcYSL3* promoter sequence and represses its expression. *PcWRKY1* inhibits Cd accumulation in the wood and bark of plants. Thus, PcWRKY1 suppresses *PcYSL3* transcription to negatively regulate Cd‐NA unloading from xylem vessels to adjacent ray parenchyma cells and its radial transport in poplar stem. The findings have provided new insights into breeding of poplars for more effective remediation of heavy metal‐contaminated soils.

## Graphical abstract

PcWRKY1 binds to the Yellow Stripe‐Like3 (*PcYSL3*) promoter region and represses *PcYSL3* expression to negatively regulate cadmium‐nicotianamine (Cd‐NA) unloading from xylem vessels to adjacent ray parenchyma cells and Cd accumulation in the phloem of *Populus* × *canescens* (Aiton) Sm.

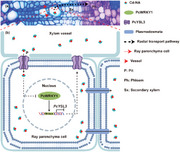

## 1 Introduction

Cadmium (Cd) is severely toxic to most organisms.^[^ ^1^ ^]^ Cd in the soil can be taken up by plants and it may eventually enter the human body through the food chain to cause various diseases.^[^ ^1^ ^]^ Thus, Cd‐contaminated soil needs to be remediated. Phytoremediation is an effective approach for remediating Cd‐contaminated soil.^[^ ^2^ ^]^ Several herbaceous plants have been identified with the capacity to accumulate high Cd concentrations in their leaves.^[^ ^3^ ^]^ However, the total amounts of Cd accumulated in herbaceous plants are limited by their low biomass. Consequently, fast‐growing woody plants, including *Populus* species, are recommended for remediating Cd‐polluted soil.^[^ ^4^ ^]^

A considerable amount of Cd can be accumulated in the aerial tissues (wood, bark, and leaves) of poplars.^[^ ^5^ ^]^ In particular, high Cd concentrations have been detected in the bark‐phloem of poplar,^[^ ^5^, ^6^ ^]^ which has not been reported previously in herbaceous plants. In general, it is assumed that Cd in the bark‐phloem can be delivered via two distinct pathways. In the first pathway, Cd in soil solution can be taken up and transported to the stele cells of roots.^[^ ^7^ ^]^ Subsequently, Cd in xylem vessels enters mesophyll cells driven by the transpiration stream and it is further transported to the bark‐phloem. Several essential elements are translocated to the bark‐phloem through this pathway.^[^ ^8^ ^]^ In the other pathway, Cd in xylem vessels of stem‐wood is radially transported to the bark‐phloem. Previous studies have shown that ray parenchyma cells adjacent to xylem vessels (also called vessel‐associated ray parenchyma cells) play key roles in transporting water, sugars, and proteins between stem‐wood and bark‐phloem.^[^ ^9^ ^]^ However, it is unclear whether one or both of these pathways contributes to Cd accumulation in the bark‐phloem of poplar.

Many transporters involved in Cd delivery have been identified in herbaceous plants, such as defensin‐like proteins, natural resistance‐associated macrophage proteins (NRAMPs), and yellow stripe‐like proteins (YSLs).^[^ ^10^ ^]^ Eight *YSL* family members have been identified in *Arabidopsis thaliana* (L.) Heynh. No previous studies have reported the involvement of these eight AtYSLs in Cd transport, but AtYSL2 mainly localizes in the vasculature of roots and leaves, and it transports iron/copper‐nicotianamine (Fe/Cu‐NA) complexes.^[^ ^11^ ^]^ ZmYS1 (the first YSL family member to be discovered) in *Zea mays* L. is also involved in transporting Cd‐2′‐deoxymugineic acid complexes into cells.^[^ ^12^ ^]^ The heterologous expression of *SnYSL3* in yeast cells indicated a role in the transport activity of Cd‐NA complexes.^[^ ^10a^ ^]^ In addition, *VcYSL6* in *Vaccinium corymbosum* L. and *BjYSL7* in *Brassica juncea* (L.) Czern. are involved in Cd transport.^[^ ^13^ ^]^ Eleven *YSL* family members have been reported in *Populus* species.^[^ ^14^ ^]^ However, no information is available regarding the involvement of YSLs in Cd transport in poplars.

WRKY proteins typically contain the WRKYGQK sequence, which can bind to the (T)TGAC(T/C) sequence (W‐box) in the promoter regions of target genes to regulate plant responses to Cd exposure.^[^ ^15^ ^]^ In *A. thaliana*, AtWRKY13 activates the expression of *Pleiotropic Drug Resistance Transporter 8*, which is involved in Cd efflux, thereby resulting in lower Cd accumulation in plants.^[^ ^16^ ^]^ Similarly, *AtWRKY12* overexpression inhibits the expression of *γ‐Glutamylcysteine Synthetase 1* and downregulates the expression of genes involved in phytochelatin synthesis to decrease the accumulation of Cd in roots and shoots.^[^ ^17^ ^]^ Recently, it was shown that *PyWRKY48* and *PyWRKY75* in *Populus yunnanensis* Dode are highly expressed in roots and their transcriptional levels are increased upon Cd exposure.^[^ ^18^ ^]^ *PyWRKY48*‐ or *PyWRKY75*‐overexpressing poplars accumulate more Cd than non‐transgenic plants.^[^ ^18^ ^]^ These findings suggest that WRKY transcription factors are essential for plant responses to Cd. About 100 *WRKY* family members have been identified in *Populus* species.^[^ ^19^ ^]^ However, it is unclear whether other *WRKY*s are involved in regulating the responses of poplar to Cd exposure.

To elucidate the pathways and underlying molecular mechanisms of Cd accumulation in the bark‐phloem of poplars, we first identified a Cd radial transport pathway involved in Cd unloading from xylem vessels to adjacent ray parenchyma cells and further radial translocation to the bark‐phloem in the stems of *Populus* × *canescens* (Aiton) Sm. We then characterized the molecular functions of a previously unrecognized PcWRKY1‐*PcYSL3* module that regulates Cd radial transport in poplar stems. We found that PcWRKY1 bound to the promoter sequence of *PcYSL3* to suppress its transcription, thereby negatively regulating Cd‐NA unloading from xylem vessels to adjacent ray parenchyma cells and its further radial translocation from the wood to the bark‐phloem in poplar stems. We elucidated the molecular mechanisms that underlie Cd radial transport in the stems of woody plants and our findings provide new insights into the breeding of poplars to more efficiently remediate heavy metal‐polluted soils.

## 2 Results

### 2.1 Cd was Radially Transported from Xylem Vessels to the Bark‐Phloem in Poplar Stems

**Figure 1:**
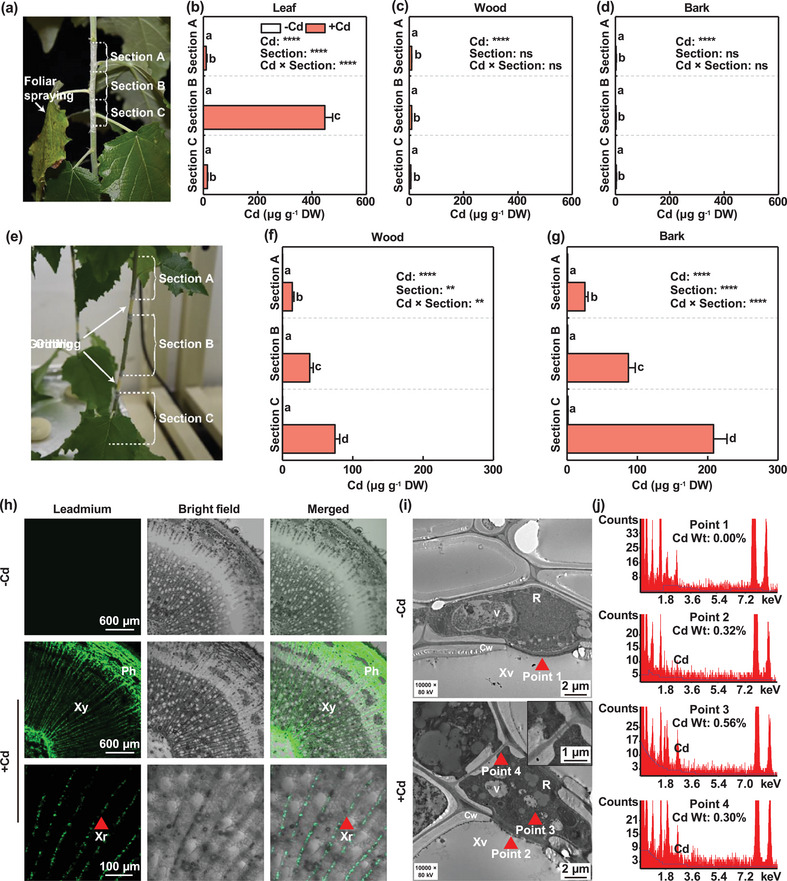
Cd radially translocated from xylem vessels to the bark‐phloem through ray parenchyma cells in *P*. × *canescens* stems. a) Schematic diagram illustrating foliar spraying of Cd and harvested shoot sections A, B, and C. b–d) Cd concentrations in the leaf (b), wood (c), and bark (d) tissues of sections A, B, and C, respectively, in panel (a) following foliar spraying with either 0 (–Cd) or 30 (+Cd) µmol L^−1^ Cd for three weeks. e) Schematic diagram illustrating stem girdling. f,g) Cd concentrations in the wood (f) and bark (g) tissues of shoot sections A, B, and C of girdled poplars exposed to either 0 (–Cd) or 50 (+Cd) µmol L^−1^ Cd for one week. h) Micrographs of stem cross sections stained with Leadmium^TM^ Green AM dye in poplars exposed to either 0 (–Cd) or 50 (+Cd) µmol L^−1^ Cd for three weeks. i) Transmission electron microscopy micrographs illustrating Cd deposits in ray parenchyma cells adjacent to xylem vessels of poplar stems exposed to either 0 (–Cd) or 50 (+Cd) µmol L^−1^ Cd for three weeks. Red triangles indicate Cd deposits. The inset shows plasmodesmata between adjacent ray parenchyma cells. j) Energy‐dispersive X‐ray spectroscopy analysis showing Cd deposits at points 1, 2, 3, and 4 in panel (i). The bars in (b), (c), (d), (f), and (g) indicate means ± standard errors (*n* = 6). Different letters on the bars in each panel indicate significant differences between treatments. *P*‐values obtained by two‐way ANOVA for Cd, section, and their interactions (Cd × Section) are also indicated. **: *P* < 0.01, ***: *P* < 0.001, ****: *P* < 0.0001, ns: not significant. Cw: cell wall, Ph: phloem, R: ray parenchyma cell, V: vacuole, Xr: xylem ray, Xv: xylem vessels, Xy: xylem.

Cd can accumulate in the bark‐phloem in woody plants, including poplars. However, it is unknown whether the Cd in bark‐phloem comes from the leaves via phloem unloading or from the xylem vessels through radial translocation. To address this question, we designed foliar spraying and stem‐girdling experiments using *P*. × *canescens* saplings (**Figure** **1**a–g). In the foliar spraying assay, poplar leaves [leaf plastochron index (LPI) = 16] were sprayed with 30 µmol L^−1^ Cd for three weeks (Figure 1a). The Cd concentration was significantly higher in the 16th leaf than the control, and it was negligible in the leaves adjacent to the 16th leaf (Figure 1b). Similarly, negligible amounts of Cd were detected in the wood and bark (Figure 1c,d). These results show that Cd in the leaves was unable to be transported to the bark and wood tissues via phloem unloading, thereby suggesting that the Cd in bark‐phloem was unlikely to have come from the leaves. In the stem‐girdling experiment, the first girdle was placed between the 14th and 15th leaves and the second between the 17th and 18th leaves, which resulted in the three stem segments (section A between the 12th and 14th leaves, section B between the 15th and 17th leaves, and section C between the 18th and 20th leaves; Figure 1e). High Cd concentrations were detected in the wood tissues in the three stem segments of girdled *P*. × *canescens* poplars after exposure to 50 µmol L^−1^ Cd for one week, but no Cd was detected in the wood tissues treated with 0 µmol L^−1^ Cd (Figure 1f). The Cd concentrations decreased gradually in the wood tissues in sections C, B, and A of girdled *P*. × *canescens* trees under Cd exposure, thereby suggesting that Cd was probably transported from the roots to the aerial tissues through the xylem vessels (Figure 1f). Similar results were obtained for the bark in these poplars (Figure 1g). Intriguingly, a high Cd concentration was found in the bark in section B, which was isolated from the bark in sections A and B by girdling (Figure 1e,g), and thus the Cd in the wood was probably radially transported to the bark of *P*. × *canescens*.

To further investigate the specific pathway that allowed Cd to be transported from the wood to the bark, we conducted histochemical and anatomic assays (Figure 1h–j). Stem cross sections of *P*. × *canescens* saplings exposed to either 0 or 50 µmol L^−1^ Cd for two weeks were stained using Leadmium™ Green AM, which can bind Cd to produce green fluorescence. No fluorescence was observed in the control, whereas intense fluorescence was found in the wood and bark tissues of Cd‐exposed *P*. × *canescens* (Figure 1h). Moreover, the fluorescence in the wood was mainly localized in xylem ray parenchyma cells (Figure 1h). In particular, intense fluorescence was observed in ray parenchyma cells adjacent to the xylem vessels (Figure 1h). To determine whether the Cd in xylem vessels was transported to its neighboring ray parenchyma cells, we prepared stem transverse sections of *P*. × *canescens* treated with either 0 or 50 µmol L^−1^ Cd for three weeks. Under transmission electron microscopy and energy‐dispersive X‐ray spectroscopy, no electron‐dense granules were found in vessel cells in the cross sections of 0 µmol L^−1^ Cd‐exposed poplars, whereas large numbers of electron‐dense granules were observed in different cells from Cd‐exposed poplars (Figure 1i). In particular, Cd in electron‐dense granules was detected at a half‐bordered pit between a xylem vessel element and ray parenchyma cell (point 2), in the cytoplasm of a ray parenchyma cell (point 3), and in the plasmodesmata between both ray parenchyma cells (point 4; Figure 1i,j). These results suggest that Cd was unloaded from xylem vessels to adjacent ray parenchyma cells and further radially translocated from the wood to the bark‐phloem in poplars.

### 2.2 PcYSL3 Active in Transport of Cd‐NA Complexes in Yeast Cells

Cd unloading from xylem vessels to adjacent ray parenchyma cells is the first step during its radial translocation from the wood to the bark‐phloem. Thus, it was essential to identify the key genes that regulated Cd unloading from the xylem vessels to adjacent ray parenchyma cells. To identify key gene candidates, we conducted transcriptomic analysis using wood tissues from *P*. × *canescens* saplings after exposure to 50 µmol L^−1^ Cd for 0, 6, 12, 36, and 72 h. The Cd concentrations gradually increased in the poplar wood tissues as the duration of Cd exposure increased (Figure S1a, Supporting Information). In addition, ca. 5702 significantly differentially expressed genes (SDEGs) were identified in the wood tissues of Cd‐exposed versus control *P*. × *canescens* (Table S1, Supporting Information). These SDEGs were categorized into 15 clusters based on their expression patterns and most were significantly enriched in six clusters (Figure S1b and Table S1, Supporting Information). In particular, the expression patterns of genes in cluster 13 (C13) corresponded well with the changes in the Cd concentrations in the wood samples (Figure S1a,b, Supporting Information), thereby indicating that key gene candidates were probably enriched in C13.

**Figure 2:**
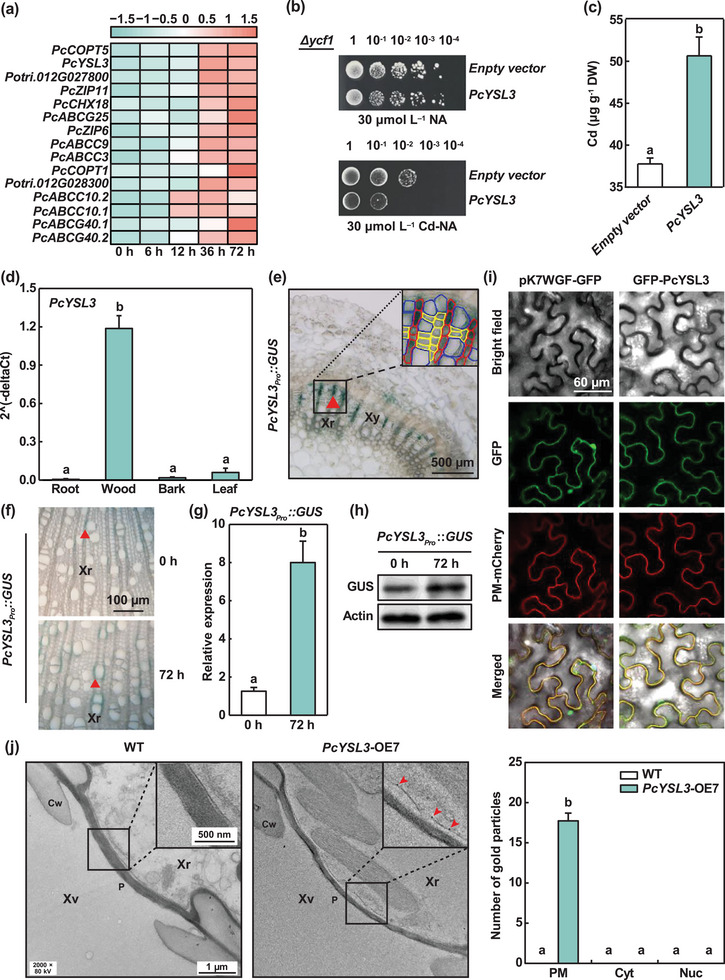
Plasma membrane‐localized PcYSL3 transported Cd‐NA from extracellular space into yeast cells. a) Expression patterns of 15 putative genes encoding metal transporters in wood of *P*. × *canescens* exposed to 50 µmol L^−1^ Cd for 0, 6, 12, 36, and 72 h. Colors indicate z‐score normalized FPKMs. Salmon color: high expression, light sea green: low expression. b) Growth status of Cd‐sensitive yeast mutant strain *Δycf1* cells transformed with either empty vector or *PcYSL3*. Yeast cells diluted to OD_600_ values of 1 to 10^−4^ were cultured on plates with galactose and either 30 µmol L^−1^ NA or Cd‐NA for four days. c) Cd concentrations in yeast cells cultured in liquid media. d) Expression levels of *PcYSL3* in different tissues of *P*. × *canescens*. e) Staining of stem cross section of *PcYSL3_Pro_::GUS* transgenic *P*. × *canescens*. Red triangle denotes a xylem ray (Xr). The inset shows ray parenchyma cells (bordered cells with red line), xylem vessels (bordered cells with blue line), and xylem fiber cells (bordered cells with yellow line). Xy: xylem. f–h) GUS activities (f), relative expression levels of *GUS* (g), and GUS protein levels (h) in stems of *PcYSL3_Pro_::GUS* transgenic poplars exposed to 50 µmol L^−1^ Cd for either 0 or 72 h. Red triangles denote xylem rays (Xr, f). The GUS protein blotting is a cropped image (h). i) Subcellular localization of PcYSL3 in tobacco leaf epidermal cells. j) Immunological detection of PcYSL3 in cross sections of *P*. × *canescens* stems immunostained using anti‐Flag antibody. Insets in WT and *PcYSL3*‐OE7 show the plasma membrane of ray parenchyma cells adjacent to the pits of xylem vessels (Xv). The expression of PcYSL3 indicated by the appearance of gold particles (red arrowheads in insert) was detected in ray parenchyma cells but not in xylem vessels. PcYSL3 was detected on the plasma membrane, but PcYSL3 was not detected in the cell wall (Cw) or pit (P). The bar graph shows the granules of PcYSL3 on the plasma membrane of ray parenchyma cells adjacent to the pits of xylem vessels in WT and *PcYSL3*‐OE7 poplars. Xr: xylem ray, PM: plasma membrane, Cyt: cytoplasm, Nuc: nucleus. The bars in (c), (d), (g), and (j) indicate means ± standard errors (*n* = 3). Different letters in each panel indicate significant differences between treatments.

To further identify key candidate genes, the SDEGs in C13 were annotated and categorized (Table S1, Supporting Information). In C13, 15 putative metal transporter genes (containing seven ATP‐binding cassette transporters) belonging to the transport category were identified and the changes in their expression levels were determined under Cd exposure (**Figure** **2**a). The sequences of these 15 genes were cloned and expressed in the Cd‐sensitive mutant yeast strain *Δycf1* (Figure S1c, Supporting Information). Similar growth performance was found in transformed yeast cells treated without Cd, but the growth of yeast cells that expressing copper transporter 5 (*PcCOPT5*) and Zrt/Irt‐like protein 11 (*PcZIP11*) was inhibited compared with those that expressing the empty vector under 30 µmol L^−1^ Cd (Figure S1c, Supporting Information). The growth of yeast cells transformed with *YSL* family member was insensitive to Cd (Figure S1c, Supporting Information), but YSL family members are able to transport Cd‐NA complexes.^[^ ^10a^ ^]^ To examine whether YSL member from *P*. × *canescens* was able to transport Cd‐NA, *Δycf1* cells that expressed *PcYSL3* were cultured in the medium containing either 30 µmol L^−1^ NA or Cd‐NA (Figure 2b). The growth of yeast cells expressing *PcYSL3* was inhibited in the medium containing Cd‐NA, and the Cd concentrations in the *PcYSL3*‐expressing yeast were significantly higher compared with those transformed with empty vector (Figure 2b,c). These results demonstrate that PcCOPT5, PcZIP11, and PcYSL3 could transport extracellular Cd or Cd‐NA into yeast cells.

The expression patterns of *PcCOPT5*, *PcZIP11*, and *PcYSL3* were analyzed in different *P*. × *canescens* tissues after exposure to 50 µmol L^−1^ Cd for 0 and 72 h (Figure 2d; Figure S2, Supporting Information). The transcriptional levels of *PcYSL3* and *PcZIP11* were significantly higher in wood than other tissues, and *PcCOPT5* was mainly expressed in the roots (Figure 2d; Figure S2a,b, Supporting Information). The transcriptional levels of *PcYSL3* increased more under Cd exposure compared with those of *PcCOPT5* and *PcZIP11* (Figure S2c–e, Supporting Information). Moreover, the results obtained by RNA in situ hybridization showed that the mRNA abundance of *PcYSL3* in ray parenchyma cells adjacent to vessels was higher compared with those of *PcCOPT5* and *PcZIP11* (Figure S2f–h, Supporting Information). These results suggest that *PcYSL3* plays a pivotal role in Cd unloading from the xylem vessels to adjacent ray parenchyma cells.

To further determine the tissue‐specific localization of *PcYSL3* and its response to Cd stress in *P*. × *canescens* wood tissues, we analyzed the expression pattern of *PcYSL3* using transgenic plants harboring the β‐glucuronidase (GUS) reporter driven by the *PcYSL3* promoter (Figure 2e–h). GUS staining was particularly intense in the ray parenchyma cells adjacent to vessels (Figure 2e), and the GUS activity was higher in cells exposed to Cd compared with those without Cd treatment (Figure 2f). In agreement, the expression level of *GUS* was upregulated by approximately eight times and the abundance of GUS protein was increased in Cd‐exposed wood (Figure 2g,h). These results suggest that PcYSL3 primarily localized in ray parenchyma cells adjacent to vessels, and its expression was significantly upregulated by Cd exposure.

To determine the subcellular localization of PcYSL3, the *GFP‐PcYSL3* recombinant vector and pMDC32‐1A CAN2b‐mCherry (a plasma membrane marker) were co‐transformed into leaf epidermal cells of *Nicotiana benthamiana* Domin. Confocal laser scanning microscopy observations demonstrated that the PcYSL3 protein localized on the plasma membrane (Figure 2i). Furthermore, immunoelectron microscopy observations showed that PcYSL3 was localized on the plasma membrane of ray parenchyma cells adjacent to vessels (Figure 2j). These results suggest that PcYSL3 was localized on the plasma membrane of ray parenchymal cells adjacent to xylem vessels and was able to translocate Cd‐NA complexes.

### 2.3 PcYSL3 Unloaded Cd‐NA from Xylem Vessels to Adjacent Ray Parenchyma Cells

**Figure 3:**
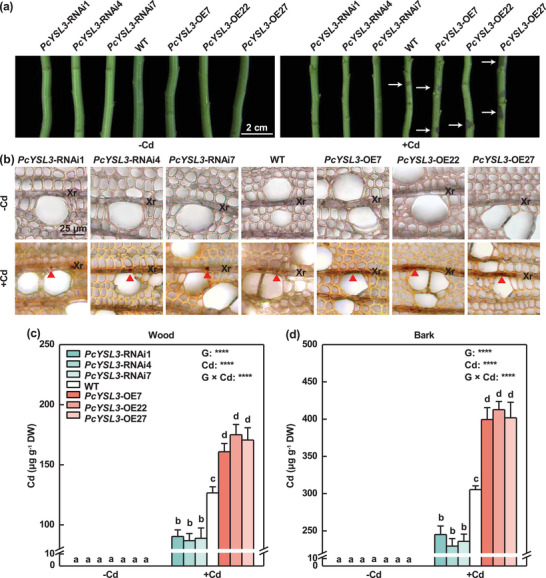
PcYSL3 mediated Cd accumulation in the xylem and phloem of *P*. × *canescens* plants treated with either 0 (–Cd) or 50 (+Cd) µmol L^−1^ Cd for four weeks. a) Phenotypes of stems of WT and *PcYSL3* transgenic plants. White arrows denote black dots. b) Cd localization in xylem ray (Xr) parenchyma cells of WT and *PcYSL3* transgenic plants. Red triangles denote Cd‐dithizone precipitation. c,d) Cd concentrations in wood (c) and bark (d) of WT and *PcYSL3* transgenic plants. The bars in (c) and (d) indicate means ± standard errors (*n* = 3). Different letters on the bars in each panel indicate significant differences between treatments. *P*‐values obtained by two‐way ANOVA for genotype (G), Cd, and their interaction (G × Cd) are also indicated. ****: *P* < 0.0001.

To investigate the function of *PcYSL3* in Cd‐NA unloading from xylem vessels to adjacent ray parenchyma cells, we generated *PcYSL3*‐knockdown and *PcYSL3*‐overexpressing *P*. × *canescens* plants (Figure S3, Supporting Information). Seven *PcYSL3*‐knockdown and seven *PcYSL3*‐overexpressing lines were obtained, and confirmed by PCR (Figure S3a,b, Supporting Information). Furthermore, three specific gene silencing lines (*PcYSL3*‐RNAi1, *PcYSL3*‐RNAi4, and *PcYSL3*‐RNAi7) with the lowest *PcYSL3* expression levels, and three *PcYSL3*‐overexpressing lines (*PcYSL3*‐OE7, *PcYSL3*‐OE22, and *PcYSL3*‐OE27) with the highest *PcYSL3* expression levels were selected for further analysis and cultured by hydroponics (Figure S3c,d, Supporting Information). The transgenic and wild‐type (WT) poplars were treated with either 0 or 50 µmol L^−1^ Cd for four weeks (Figure S3e, Supporting Information). No differences in growth performance were observed between *PcYSL3*‐transgenic and WT poplars without Cd exposure (**Figure** **3**a; Figure S3e, Supporting Information). However, when exposed to Cd, fewer black spots, which are a symptom of Cd toxicity, developed on the stems of *PcYSL3*‐knockdown poplars than WT plants, and more black spots were found on the stems of *PcYSL3*‐overexpressing poplars compared with WT plants (Figure 3a).

Cd staining was conducted using dithizone in *P*. × *canescens* wood (Figure 3b). No Cd was found in the wood of *PcYSL3*‐transgenic and WT poplars treated with 0 µmol L^−1^ Cd (Figure 3b). Less Cd was observed in the ray parenchyma cells of *PcYSL3*‐knockdown poplars than WT plants, and more Cd was found in the cells of *PcYSL3*‐overexpressing poplars compared with WT plants when exposed to 50 µmol L^−1^ Cd (Figure 3b). Cd was not detected in the wood and bark tissues of *PcYSL3*‐transgenic and WT poplar plants treated with 0 µmol L^−1^ Cd (Figure 3c,d). When exposed to 50 µmol L^−1^ Cd, the Cd concentrations were 30% and 23% lower in the wood and bark tissues of *PcYSL3*‐knockdown poplars, respectively, than WT plants, whereas they were ca. 30% higher in *PcYSL3*‐overexpressing versus WT poplars under Cd exposure (Figure 3c,d). To investigate whether Cd was unloaded from xylem vessels to adjacent ray parenchyma cells via PcYSL3 in the form of Cd‐NA complexes, we analyzed the NA concentrations and Cd‐NA translocation in wood tissues of *PcYSL3*‐transgenic and WT poplars (Figure S4, Supporting Information). The NA concentrations were significantly lower in the wood of *PcYSL3*‐knockdown plants than WT poplars, but markedly higher in the wood of *PcYSL3*‐overexpressing poplars compared with those in WT plants without Cd exposure (Figure S4a, Supporting Information). The NA concentrations were higher in *PcYSL3*‐overexpressing poplars compared with those in WT plants when exposed to 50 µmol L^−1^ Cd (Figure S4a, Supporting Information). The aerial tissues of *PcYSL3*‐transgenic and WT poplars were cultivated with either 30 µmol L^−1^ NA or Cd‐NA for 1 h, and cross sections of the 8th internode were obtained and stained with Leadmium™ Green AM (Figure S4b, Supporting Information). No fluorescence was detected in *PcYSL3*‐transgenic and WT poplars under NA exposure (Figure S4b, Supporting Information). However, under Cd‐NA exposure, weaker green fluorescence was observed in the ray parenchyma cells adjacent to xylem vessels in *PcYSL3*‐knockdown poplars than WT plants, and more intense green fluorescence was found in the cells of *PcYSL3*‐overexpressing poplars compared with WT plants (Figure S4b, Supporting Information). These results suggest that PcYSL3 facilitated the unloading of Cd‐NA complexes from xylem vessels to adjacent ray parenchyma cells.

### 2.4 PcWRKY1 Bound to the Promoter Region of *PcYSL3* and Inhibited its Expression

**Figure 4:**
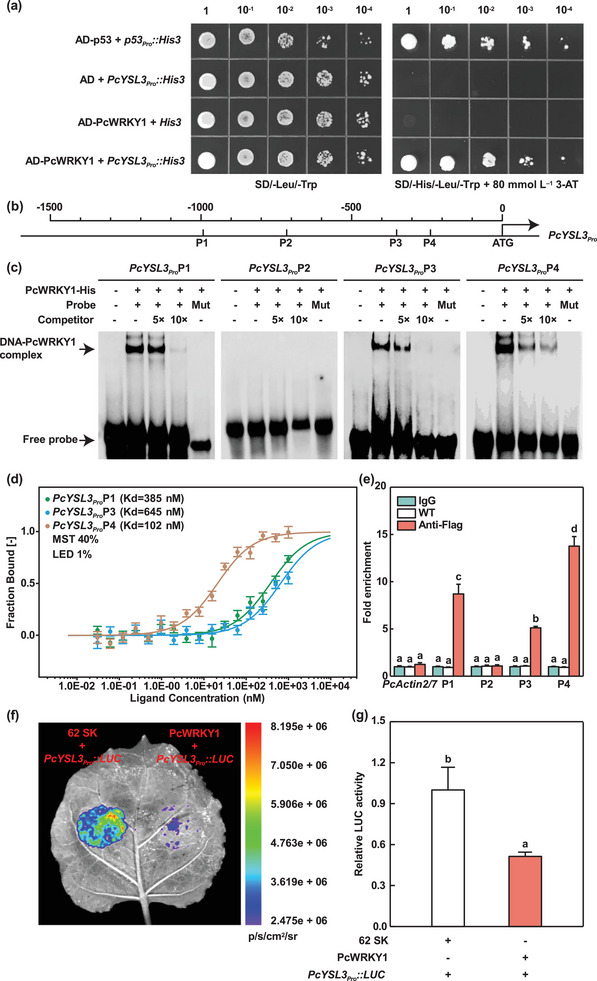
PcWRKY1 bound directly to the promoter of *PcYSL3* and inhibited its expression. a) Yeast one‐hybrid assays demonstrated the interaction between PcWRKY1 and the promoter of *PcYSL3* in yeast cells. Yeast cells that co‐expressed plasmids were plated on either SD/‐Leu/‐Trp or SD/‐His/‐Leu/‐Trp containing glucose. b) Schematic diagram showing the structure of the *PcYSL3* promoter. The fragment positions (P1, P2, P3, and P4) indicate the sites containing W‐boxes in the *PcYSL3* promoter. Four fragments used for EMSA, MST and ChIP‐qPCR assays are indicated. c) EMSA demonstrated that PcWRKY1 bound to P1, P3, and P4 sites of *PcYSL3* promoter sequence. The biotin‐labeled probe incubated with PcWRKY1‐His protein was tested (lane 2). Biotin‐labeled probe incubated with PcWRKY1‐His protein alone was used as a negative control (lane 1). Competitive probes of 5× and 10× (lacking biotin label) were also used (lanes 3 and 4, respectively). Mut: mutant probe. d) MST assays showed that PcWRKY1 bound to P1, P3, and P4. Fraction Bound: baseline corrected fluorescence normalized for amplitude. e) ChIP‐qPCR assay results showing the direct interaction between PcWRKY1 and the *PcYSL3* promoter. Chromatin from WT and *PcWRKY1* overexpressing poplars was immunoprecipitated using anti‐Flag antibodies. IgG was used as a negative control, and *PcActin2/7* as a reference. f) Dual‐luciferase assays in *N. benthamiana* leaves, where 62 SK+*PcYSL3_Pro_::LUC* was used as the control. Luminescence images were recorded 3 days after infiltration. g) The activities of LUC and REN were measured in sequence, and the LUC/REN ratio was calculated as the final transcriptional activity. The bars in (d), (e), and (g) indicate means ± standard errors (*n* = 3). Different letters in each panel indicate significant differences compared with the control.

To investigate whether upstream transcription factors regulate the expression of *PcYSL3* to mediate Cd‐NA transport, we constructed a gene regulatory network (GRN) according to the expression patterns of SDEGs (Figure S5a and Table S2, Supporting Information). After analyzing the possibility of binding between transcription factors and the *PcYSL3* promoter sequence, we selected PcWRKY1 for further investigation (Figure S5b,c, Supporting Information). The results obtained in yeast one‐hybrid assays demonstrated that PcWRKY1 bound to the sequence of the *PcYSL3* promoter region (**Figure** **4**a). PcWRKY1 belongs to the WRKY family, which usually bind to the W‐box sequences in target genes. Four W‐box sequences (P1, P2, P3, and P4) were found in the promoter region of *PcYSL3* (Figure 4b).

Electrophoretic mobility shift assays (EMSA) were conducted to identify the specific regions in the *PcYSL3* promoter sequence bound by PcWRKY1 (Figure 4c). Obviously, PcWRKY1 physically interacted with DNA fragments from the P1, P3, and P4 sites in the *PcYSL3* promoter sequence, but not with those from the P2 site (Figure 4c). Moreover, mutating the W‐box sequence completely abolished the binding of PcWRKY1 protein to the probe sequences (Figure 4c). To further determine the binding strength of PcWRKY1 to the four sites in the *PcYSL3* promoter sequence, we performed microscale thermophoresis (MST) assays (Figure 4d; Figure S6, Supporting Information). In vitro, glutathione S‐transferase (GST) protein was unable to bind to any of the P1, P2, P3, and P4 sites, but the PcWRKY1‐GST fusion protein had high to low binding capacities with the P4, P1, and P3 sites in the *PcYSL3* promoter region (Figure 4d; Figure S6a,b, Supporting Information). In addition, chromatin immunoprecipitation (ChIP)‐qPCR analysis of WT and *PcWRKY1*‐Flag (*PcWRKY1*‐OE2, ‐OE6, and ‐OE7) poplars using specific primers (Table S3, Supporting Information) demonstrated that PcWRKY1 bound to the *PcYSL3* promoter fragments (P1, P3, and P4) pulled down by anti‐Flag antibodies in *PcWRKY1*‐Flag lines, but not in WT poplars (Figure 4e). We conducted a dual‐luciferase activity assay to examine the effects of PcWRKY1 on regulating the expression of *PcYSL3* (Figure 4f,g). The relative firefly luciferase (LUC) activity was significantly lower in the leaves of tobacco plants co‐infiltrated with PcWRKY1 + *PcYSL3_Pro_::LUC* compared with the control (Figure 4f,g). These results suggest that PcWRKY1 repressed *PcYSL3* transcription by binding directly to its promoter sequence.

### 2.5 Expression Patterns of *PcWRKY1* and Localization of Protein

**Figure 5:**
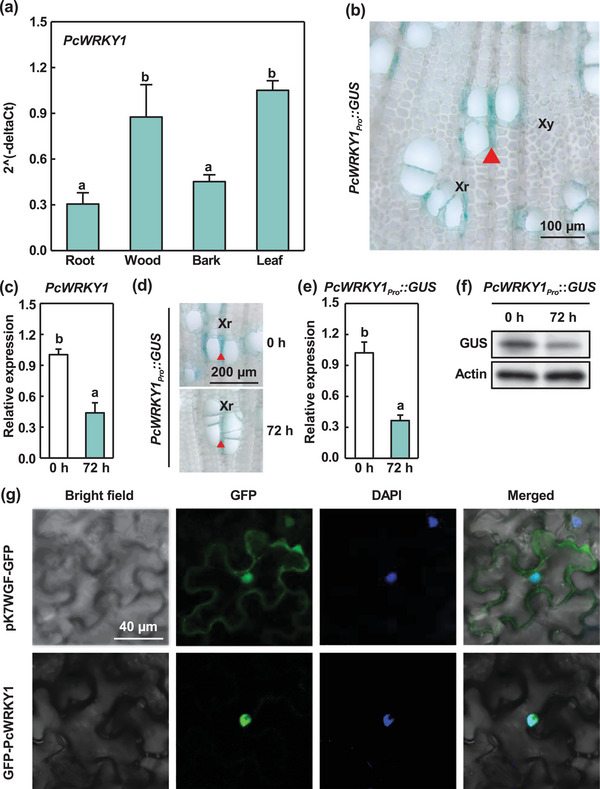
Expression patterns of *PcWRKY1* and subcellular localization of PcWRKY1 protein. a) Expression levels of *PcWRKY1* in different *P*. × *canescens* tissues. b) Staining of xylem cross section in *PcWRKY1_Pro_::GUS* transgenic *P*. × *canescens* plants. The red triangle denotes a xylem ray (Xr). Xy: xylem. c) Expression levels of *PcWRKY1* in wood exposed to 50 µmol L^−1^ Cd for either 0 or 72 h. d–f) GUS activities (d), relative expression levels of *GUS* (e), and GUS protein levels in stems of *PcWRKY1_Pro_::GUS* transgenic poplars exposed to 50 µmol L^−1^ Cd for either 0 or 72 h. The red triangles denote xylem rays (Xr, d). The GUS protein blotting is a cropped image (f). g) Subcellular localization of PcWRKY1 in tobacco leaf epidermal cells. The bars in (a), (c), and (e) indicate means ± standard errors (*n* = 3). Different letters in each panel indicate significant differences between treatments.

Our results demonstrated that PcWRKY1 repressed the expression of *PcYSL3* in ray parenchyma cells in *P*. × *canescens* stems. Thus, our findings indicated that *PcWRKY1* was highly expressed in poplar wood and its expression in response to Cd exposure was opposed to that of *PcYSL3*. To test this assumption, we analyzed the expression patterns of *PcWRKY1* and its expression under Cd exposure in *P*. × *canescens* (**Figure** **5**a–f). The expression levels of *PcWRKY1* were significantly higher in wood and leaf tissues than those in root and bark tissues (Figure 5a). Furthermore, GUS staining showed that PcWRKY1 localized in ray parenchyma cells adjacent to xylem vessels (Figure 5b). The expression of *PcWRKY1* was repressed in the wood of *P*. × *canescens* under Cd treatment (Figure 5c), which was confirmed by GUS staining of *PcWRKY1_Pro_::GUS* poplars, the quantification of the *GUS* relative expression levels using quantitative reverse transcription PCR (qRT‐PCR), and the detection of GUS protein abundances (Figure 5d–f). To determine the subcellular localization of PcWRKY1, the GFP‐PcWRKY1 fusion protein was transiently expressed in *N. benthamiana* leaf epidermal cells and PcWRKY1 localized in nuclei (Figure 5g). These results suggest that the expression level of *PcWRKY1* was inhibited by Cd exposure and its protein was localized in the nuclei of ray parenchyma cells adjacent to xylem vessels.

### 2.6 PcWRKY1 Inhibited Cd Transport from Wood to Bark‐Phloem

To investigate the roles of *PcWRKY1* in Cd transport from the wood to the bark‐phloem in poplars, *PcWRKY1*‐knockdown and *PcWRKY1*‐overexpressing *P*. × *canescens* plants were generated (Figure S7, Supporting Information). Eight *PcWRKY1*‐knockdown and six *PcWRKY1*‐overexpressing lines were identified by PCR (Figure S7a,b, Supporting Information). Moreover, three *PcWRKY1*‐specific gene silencing lines (*PcWRKY1*‐RNAi3, *PcWRKY1*‐RNAi4, and *PcWRKY1*‐RNAi6) with the lowest *PcWRKY1* expression levels, and three *PcWRKY1*‐overexpressing lines (*PcWRKY1*‐OE2, *PcWRKY1*‐OE6, and *PcWRKY1*‐OE7) with the highest *PcWRKY1* expression levels were selected and cultured by hydroponics (Figure S7c–e, Supporting Information). The mRNA levels of *PcYSL3* were also analyzed in *PcWRKY1*‐transgenic poplars (Figure S7f, Supporting Information). The transcript levels of *PcYSL3* were significantly higher in *PcWRKY1*‐knockdown poplars whereas the opposite was found in *PcWRKY1*‐overexpressing plants, irrespective of Cd treatments (Figure S7f, Supporting Information).

**Figure 6:**
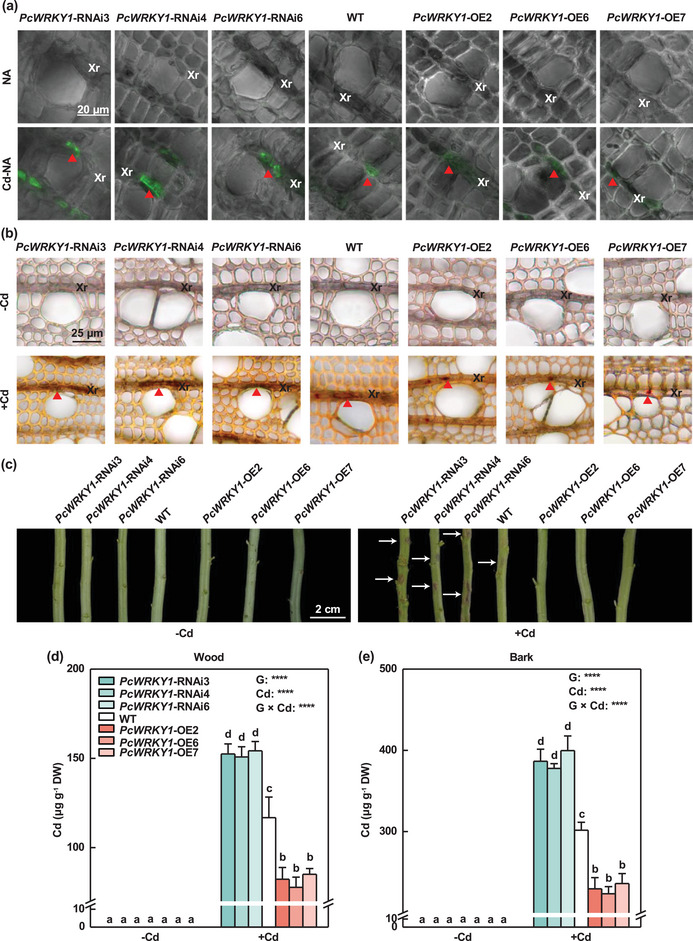
PcWRKY1 mediated the accumulation of Cd in the xylem and phloem of *P*. × *canescens*. a) Micrographs of xylem cross sections in WT and *PcWRKY1* transgenic poplars exposed to either 30 µmol L^−1^ NA or Cd‐NA for 1 h. Red triangles denote Cd‐Leadmium. b) Cd localization in xylem ray (Xr) parenchyma cells of WT and *PcWRKY1* transgenic plants treated with either 0 (–Cd) or 50 (+Cd) µmol L^−1^ Cd for four weeks. Red triangles denote Cd‐dithizone precipitation. c) Phenotypes of stems of WT and *PcWRKY1* transgenic plants treated with either 0 (–Cd) or 50 (+Cd) µmol L^−1^ Cd for four weeks. White arrows denote black dots. d,e) Cd concentrations in wood (d) and bark (e) of WT and *PcWRKY1* transgenic plants treated with either 0 (–Cd) or 50 (+Cd) µmol L^−1^ Cd for four weeks. The bars in (d) and (e) indicate means ± standard errors (*n* = 3). Different letters on bars in each panel indicate significant differences between treatments. *P*‐values according to two‐way ANOVA based on genotype (G), Cd, and their interaction (G × Cd) are also indicated. ****: *P* < 0.0001.

Assays were performed using *PcWRKY1*‐transgenic and WT poplars in a similar manner to the experiment using *PcYSL3*‐transgenic *P*. × *canescens* cultivated with either 30 µmol L^−1^ NA or Cd‐NA (**Figure** **6**a). More intense green fluorescence was detected in the ray parenchyma cells of *PcWRKY1*‐knockdown poplars than WT plants, whereas the opposite was found in *PcWRKY1*‐overexpressing poplars (Figure 6a).

Cd was not found in *PcWRKY1*‐transgenic and WT poplars without Cd treatment (Figure 6b). However, under treatment with 50 µmol L^−1^ Cd, more Cd was found in the wood of *PcWRKY1*‐knockdown poplars compared with WT, whereas the opposite was observed in *PcWRKY1*‐overexpressing poplars (Figure 6b). No difference was observed in growth between *PcWRKY*‐transgenic and WT poplars irrespective of Cd treatment (Figure S7e, Supporting Information). Cd exposure inhibited the growth of *PcWRKY1*‐transgenic and WT poplars (Figure S7e, Supporting Information). Under exposure to Cd, the stems of *PcWRKY1*‐knockdown poplars had more black spots than WT plants, but no black spots were found on the stems of *PcWRKY1*‐overexpressing poplars (Figure 6c). In addition, Cd was not detected in the wood and bark tissues of *PcWRKY1*‐transgenic and WT poplars without Cd exposure (Figure 6d,e). Under 50 µmol L^−1^ Cd treatment, the Cd concentrations were 34% and 28% higher in the wood and bark tissues of *PcWRKY1*‐knockdown poplars, respectively, than WT plants, but they were markedly lower in *PcWRKY1*‐overexpressing plants compared with WT plants exposed to Cd (Figure 6d,e). These results suggest that PcWRKY1 inhibited Cd translocation from wood to bark‐phloem.

## 3 Discussion

Compared with the high amounts of Cd that accumulate in the leaves of most herbaceous hyperaccumulators,^[^ ^3^ ^]^ more Cd accumulated in the bark‐phloem of *P*. × *canescens*,^[^ ^5^, ^6^ ^]^ which is a unique feature and essential for Cd accumulation in the aerial tissues of woody plants. However, the pathway that allows poplars to transport Cd in the wood to the bark‐phloem and the molecular mechanisms involved in this process are unclear. Therefore, in the present study, we analyzed the Cd concentrations and associated transport pathways in the leaf, wood, and bark tissues of *P*. × *canescens* using assays based on foliar Cd spraying and girdling, and Cd staining and transmission electron microscopy observations. We found that PcWRKY1 repressed the transcription of *PcYSL3* to negatively regulate Cd radial transport from the wood to the bark‐phloem in poplars based on molecular analyses.

### 3.1 Cd Radially Transported from the Wood to Bark‐Phloem via Ray Parenchyma Cells in *P*. × *Canescens*

Xylem ray parenchyma cells adjacent to vessels are usually connected to the xylem vessels through half‐bordered pits on the cell walls.^[^ ^9c^ ^]^ The pit membrane at the center of the pit is highly permeable to water.^[^ ^20^ ^]^ Water that crosses the pit membranes can be further transported from the apoplast to the symplast via transporters localized on the plasma membrane of ray parenchyma cells.^[^ ^21^ ^]^ Subsequently, water in ray parenchyma cells can be translocated from the xylem to phloem through plasmodesmata between ray parenchyma cells.^[^ ^22^ ^]^ Experimental studies have shown that the pit membrane is also permeable to lanthanum nitrate.^[^ ^20^ ^]^ In *A. thaliana*, sodium ions that cross the pit membranes are unloaded from the vessels to xylem ray parenchyma cells.^[^ ^23^ ^]^ Proteins in ray parenchyma cells can be translocated from the xylem to phloem in stems of *P. nigra* L.^[^ ^9b^ ^]^ To the best of our knowledge, no previous studies have identified other solutes involved in this translocation pathway. In the present study, for the first time, we showed that Cd can be radially transported from the wood to the bark‐phloem via ray parenchyma cells.

In the present study, the foliar Cd spraying assay demonstrated that foliar Cd could not be translocated to the bark via phloem unloading in poplars. In poplar leaves, Cd is mainly localized in the veins and adjacent intercellular spaces.^[^ ^5^ ^]^ The accumulation of Cd in leaf veins leads to increases in the amount of tannin in the xylem and cells necrosis in the veins,^[^ ^24^ ^]^ which can limit the redistribution of Cd from the leaves to other tissues. Therefore, only a negligible amount of Cd was detected in the wood and bark tissues of poplars exposed to foliar Cd spraying. By contrast, the stem‐girdling experiment demonstrated that Cd was radially transported from the wood to the bark‐phloem. Cd staining and transmission electron microscopy observations of poplar stem cross sections further showed that similar to the translocation of water from xylem vessels to the bark‐phloem, Cd was unloaded from xylem vessels to adjacent ray parenchyma cells via transporters on the plasma membrane of these ray parenchyma cells and then radially transported to the bark‐phloem through plasmodesmata between ray parenchyma cells.

### 3.2 PcWRKY1‐*PcYSL3* Module Mediates Cd‐NA Unloading from Xylem Vessels to Adjacent Ray Parenchyma Cells in *P*. × *Canescens*

Several transporters, including OsNRAMP5 in *Oryza sativa* L., heavy metal transporting ATPase 2 in *Brassica parachinensis* L.H. Bailey, and cadmium‐induced protein AS 8 in *P. euphratica* Oliv., contribute to Cd transport into plant cells,^[^ ^25^ ^]^ but no transporters have been previously reported to play roles in Cd unloading from xylem vessels to ray parenchyma cells. In the present study, for the first time, we identified PcYSL3 as a key transporter involved in Cd‐NA unloading from xylem vessels to adjacent ray parenchyma cells. YSL proteins belong to the oligopeptide transporter superfamily, and most YSLs function as metal‐NA carriers, playing key roles in metal ion homeostasis in plants.^[^ ^26^ ^]^ In *Solanum nigrum* L., SnYSL3 protein (a homolog of PcYSL3 sharing 76% similarity at the amino acid level) has a Cd‐NA transport activity, and *SnYSL3*‐overexpressing *A. thaliana* plants exhibited enhanced Cd transport from the roots to shoots.^[^ ^10a^ ^]^ Similarly, *VcYSL6‐* or *BjYSL7*‐overexpressing tobacco plants had higher Cd levels in the aerial tissues than WT plants.^[^ ^13^ ^]^ RNA in situ hybridization and staining of *PcYSL3_Pro_::GUS* transgenic plants showed that PcYSL3 was highly expressed in ray parenchyma cells adjacent to xylem vessels in *P*. × *canescens*. Interestingly, aquaporins involved in water transport across the plasma membrane, including plasma membrane intrinsic protein 2;3 and tonoplast intrinsic protein 2;1, are also highly expressed in ray parenchyma cells adjacent to xylem vessels in *P*. × “Okanese”, contributing to radial water flow from the xylem to the cambial region.^[^ ^21^ ^]^ Subcellular localization and immunoelectron microscopy observations showed that PcYSL3 localized on the plasma membrane, which is similar to the subcellular localization of SnYSL3 in *S. nigrum*.^[^ ^10a^ ^]^ In addition, genetic analyses demonstrated that *PcYSL3*‐knockdown poplars accumulated less Cd in the wood (especially in ray parenchyma cells) and bark tissues than WT plants, whereas the opposite results were found in *PcYSL3*‐overexpressing lines, which agrees with previous findings obtained for *SnYSL3*‐overexpressing *A. thaliana* plants treated with Cd.^[^ ^10a^ ^]^ Moreover, functional complementation in Cd‐sensitive yeast mutant strain *Δycf1* cells and Cd fluorescent staining in *PcYSL3* transgenic poplars demonstrated that PcYSL3 unloaded Cd‐NA complexes from xylem vessels to adjacent ray parenchyma cells. Similarly, SnYSL3 also transports Cd‐NA complexes in yeast cells.^[^ ^10a^ ^]^ Currently, there is no experimental evidence supporting the reciprocal proteins of YSL3 that work together in plants in response to Cd exposure. HvYS1 of *Hordeum vulgare* L., which belongs to the same YSL family as PcYSL3, forms a homodimer to transport substrates.^[^ ^27^ ^]^ Since PcYSL3 shares a similar structural domain to HvYS1, it is probable that PcYSL3 also forms a homodimer to transport Cd‐NA complexes, which needs to be examined in future studies. Overall, these results suggest that the plasma membrane‐localized PcYSL3 is highly expressed in ray parenchyma cells adjacent to xylem vessels and it facilitates the unloading of Cd‐NA complexes from xylem vessels to adjacent ray parenchyma cells.

WRKY transcription factors play critical roles in plant responses to abiotic stresses.^[^ ^15^ ^]^ In particular, several WRKY transcription factors have been shown to function as key regulators of Cd accumulation in plants.^[^ ^16^, ^17^ ^]^ For instance, ThWRKY7 in *Tamarix hispida* Willd. interacts with the promoter of *V‐ATPase c subunit 1* to activate its transcription and reduce the accumulation of Cd in plants.^[^ ^28^ ^]^ GmWRKY142 in *Glycine max* (L.) Merr. reduces the uptake of Cd through the roots by binding directly to the promoters of *Cadmium Tolerance 1‐1* (*GmCDT1‐1*) and *GmCDT1‐2*, and activating their expression.^[^ ^29^ ^]^ AtWRKY12 and AtWRKY33 can negatively regulate Cd accumulation in *A. thaliana*.^[^ ^17^, ^30^ ^]^ Similarly, we found that PcWRKY1 negatively regulated the accumulation of Cd in the wood and bark of poplar stems. No previous studies have reported the effects of the interactions between WRKY and YSL members on Cd accumulation in plants. However, it was recently shown that AtWRKY12 binds to the promoters of *AtYSL1* and *AtYSL3* to repress their expression and negatively regulate iron accumulation in Arabidopsis seeds.^[^ ^31^ ^]^ In this study, the results of the yeast one‐hybrid, EMSA, MST, ChIP‐qPCR and LUC experiments demonstrated that PcWRKY1 directly binds to the *PcYSL3* promoter region and represses *PcYSL3* transcription. In the future studies, chromatin immunoprecipitation sequencing is important to further identify more PcWRKY1 target genes which regulate Cd radial transport. It will also be critical to create the transgenic poplars with knockdown *PcYSL3* in the background of *PcWRKY1* overexpression to provide genetic evidence for PcWRKY1‐*PcYSL3* module mediating regulation of Cd radial transport.

The qRT‐PCR analysis revealed that the expression of *PcWRKY1* was downregulated by Cd exposure. Consistently, GUS staining, together with the detection of *GUS* expression levels and GUS protein abundance, demonstrated that *PcWRKY1* promoter activity was also inhibited by Cd exposure. Future studies should assess PcWRKY1 protein levels to confirm whether its expression is suppressed by Cd treatment. Interestingly, our data has revealed that eight transcription factors were positioned the upstream of *PcWRKY1* in the GRN. It is likely that ethylene response factor 1 (PcERF1) and PcERF4 were potential regulators of *PcWRKY1* due to the presence of *cis*‐elements (AGCCGCC) in the promoter sequence of *PcWRKY1* recognized by PcERF1 and PcERF4. By contrast, the expression of *PcERF1* and *PcERF4* was significantly upregulated in the wood of *P*. × *canescens* exposed to Cd. These results indicate PcERF1 and/or PcERF4 might negatively regulate the transcription of *PcWRKY1* in the Cd‐treated wood. Similarly, AtERF1 and AtERF4 have been demonstrated to act as transcription repressors in *A. thaliana*.^[^ ^32^ ^]^ In future studies, functional characterization of these two regulators will be helpful for us to better understand the upstream regulation of PcWRKY1‐*PcYSL3* module underlying Cd accumulation in bark‐phloen of poplars.

Overall, these results suggest that PcWRKY1 binds to the *PcYSL3* promoter sequence to suppress its expression and negatively regulate Cd‐NA unloading from xylem vessels to adjacent ray parenchyma cells in poplar stems.

**Figure 7:**
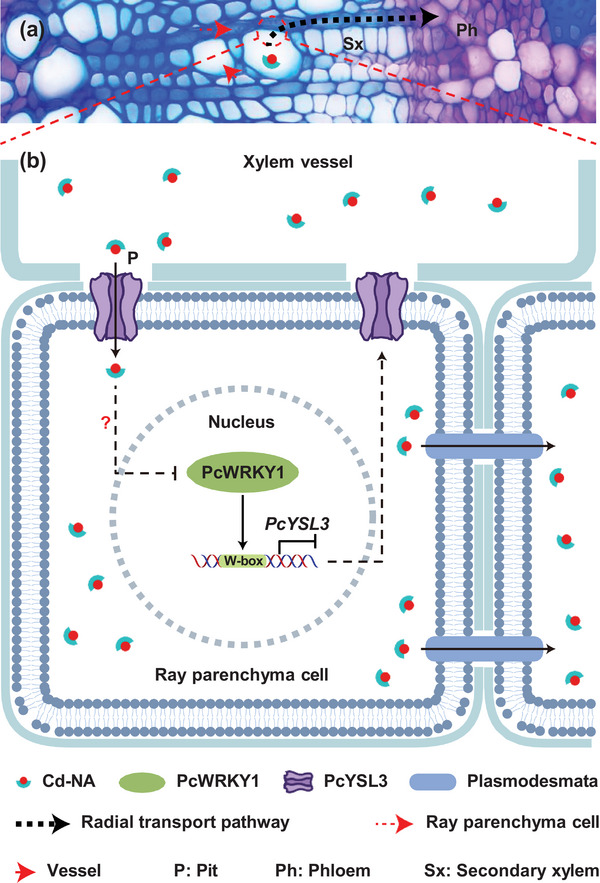
Schematic model illustrating the role of PcWRKY1 in the regulation of *PcYSL3* involved in Cd‐NA unloading from xylem vessels to adjacent ray parenchyma cells and further radial transport to the bark‐phloem. a) Cross section of *P*. × *canescens* stem where the red circle shows a ray parenchyma cell adjacent to the xylem vessel. b) Role of PcWRKY1 in regulating *PcYSL3* in Cd radial transport. PcWRKY1 binds to the *PcYSL3* promoter region and represses the expression of *PcYSL3* to negatively regulate Cd‐NA unloading from xylem vessels to adjacent ray parenchyma cells and Cd accumulation in the phloem. The question mark indicates an unknown signal.

As summarized in **Figure** **7**, a Cd radial transport pathway in poplar stems and a previously unrecognized PcWRKY1‐*Yellow Stripe‐Like 3* (*PcYSL3*) module regulating Cd transport were identified in *P*. × *canescens*. The Cd transport pathway from the wood to the bark‐phloem was identified in *P*. × *canescens*, in which Cd‐NA was unloaded from xylem vessels into adjacent ray parenchyma cells, and then radially transported to the bark‐phloem. The plasma membrane‐localized *PcYSL3* was highly expressed in ray parenchyma cells adjacent to xylem vessels and PcYSL3 protein facilitated the transport of Cd‐NA complexes from xylem vessels to adjacent ray parenchyma cells. The Cd‐NA complexes enter the ray parenchyma cells and repress *PcWRKY1* transcription through an unknown signal. PcWRKY1 directly bound to the *PcYSL3* promoter and inhibited its expression to negatively regulate Cd accumulation in the wood and bark of *P*. × *canescens* when exposed to Cd. These results suggest that PcWRKY1 suppresses the transcription of *PcYSL3* to negatively regulate Cd radial transport from the stem‐wood to the bark‐phloem in poplars.

## 4 Experimental Section

### Plant Materials and Treatments

*Populus* × *canescens* (Aiton) Sm. (syn. *P. tremula* L. × *P. alba* L. 717‐1B4) plantlets were generated by micropropagation,^[^ ^33^ ^]^ and cultured in a climate chamber (day/night temperature: 25 °C/18 °C, relative air humidity: 50–60%, light per day: 16 h, and photosynthetic photon flux: 150 mmol m^−2^ s^−1^). After four weeks, the rooted plants were transplanted into 10‐L pots filled with sand. Each plant was provided with 50 mL of one‐fourth Hoagland solution every two days for 40 days. Subsequently, the plants were transferred to a hydroponic system and cultivated in a greenhouse.^[^ ^34^ ^]^ A foliar Cd spraying assay was performed to examine whether Cd could be transported from the leaves to the wood and bark in *P*. × *canescens*. The 16th leaf of each poplar (LPI = 16) was marked and the stem region between the 15th and 17th leaves was wrapped with Parafilm M^®^ to avoid Cd contamination during Cd foliar spraying. The 16th leaf was sprayed with 5 mL of either 0 or 30 µmol L^−1^ CdCl_2_ containing 0.1% Tween‐20 at 8:00, 14:00, and 20:00 each day. After three weeks, the 15th, 16th, and 17th leaves, and the corresponding stem segments (each segment contained the stem 1.5 cm above and below the node) were separated. The wood (also called secondary xylem) and bark were further separated from each stem segment. The separated leaves were washed in EDTA‐Na_2_ solution (20 mmol L^−1^) for 5 min and subsequently in distilled water three times. The harvested materials were immediately frozen in liquid nitrogen and stored at –80 °C for further analysis.

### Determination of Cd in Samples

Cd concentrations were determined in the wood, bark, and leaf tissues using inductively‐coupled plasma mass spectrometry (7700X, Agilent Technologies, Santa Clara, USA), as described in the previous study.^[^ ^35^ ^]^

### Cd Distribution in Cross Sections of Stems

Poplars were treated with either 0 or 50 µmol L^−1^ CdCl_2_ for two weeks to determine the distribution of Cd in the cross sections of stems. The 21st internode (ca. 2 cm) was then harvested and stained with 2 mL 100 µg mL^−1^ of Leadmium™ Green AM (A10024, Invitrogen, Paisley, UK) for 2 h in the dark. Cross sections of the stained internodes were obtained by using a cryostat microtome (CM1950, Leica Biosystems, Wetzlar, Germany). The transverse sections were observed by confocal laser scanning microscopy (LSM880, Carl Zeiss, Jena, Germany) with green fluorescence excitation/emission wavelengths of 488 nm/515 to 535 nm, respectively.

### Subcellular Cd Localization

To characterize the subcellular localization of Cd, poplars were treated with either 0 or 50 µmol L^−1^ CdCl_2_ for three weeks. The 21st internode (ca. 2 cm) was then harvested and cut into cubes (ca. 1 mm^3^). These cubes were prepared and observed by transmission electron microscopy (HT7700, Hitachi, Tokyo, Japan), as described in the previous study.^[^ ^5^ ^]^ The Cd concentrations at different positions in the samples were estimated by energy‐dispersive X‐ray spectroscopy.^[^ ^5^ ^]^

### Total RNA Isolation, RNA Sequencing, and Data Analysis

To identify key genes involved in Cd transport from the stem‐wood to the bark‐phloem, poplars were exposed to 50 µmol L^−1^ CdCl_2_ for 0, 6, 12, 36, and 72 h. Stem‐wood samples were then harvested. A subsample of wood was used to determine the Cd concentration as described above. Total RNA was isolated from the remaining wood samples using a total RNA purification kit (TRK1001, LianChuan Science, Hangzhou, China) according to the manufacturer's instructions. Sequencing libraries were generated using an RNA library preparation kit (E7503, New England Biolabs, Ipswich, USA) and sequenced with a Novaseq 6000 system (Novogene, Tianjin, China). Raw sequence data were trimmed and clean reads were obtained. Clean reads were mapped to the reference genome of *P*. × *canescens* (https://www.aspendb.org/databases/spta‐717‐genome, 28 July 2021) using HISAT2 (version 2.2.1). The raw sequence data were submitted to the National Genomics Data Center (https://ngdc.cncb.ac.cn) under BioProject (Project ID: PRJCA023618). Three biological replicates were collected and sequenced for each time point. Gene expression levels were quantified by employing the fragments per kilobase of exon model per million mapped reads (FPKM). The differential expression levels of genes were calculated based on the normalized FPKM as described in a previous study.^[^ ^36^ ^]^ Moreover, the fold changes in differentially expressed genes (DEGs) were determined using the FPKMs of genes under Cd treatment for 6, 12, 36, and 72 h divided by those under Cd treatment for 0 h. DEGs with *P*‐values less than 0.05 and absolute fold change values greater than 2 were considered significant DEGs. Significant DEGs were further classified based on their expression patterns as described previously.^[^ ^37^ ^]^ Subsequently, the identifiers of significant DEGs in *P*. × *canescens* were submitted to MapMan (https://mapman.gabipd.org/, 8 October 2021) for functional category analysis.

### Cloning Full‐Length cDNAs of Genes and Yeast Expression Assays

To test the functions of selected genes in Cd transport, the full‐length coding sequences (CDSs) of these genes were cloned and expressed in *Saccharomyces cerevisiae* Meyen ex E.C. Hansen mutant strain *Δycf1* (*MATα ura3‐52 his6 leu2‐3,‐112 his3‐Δ200 trp1‐901 lys2‐801 suc2‐Δ, ycf1::hisG*), as described in a previous study.^[^ ^35^ ^]^ Briefly, full‐length cDNAs of 15 genes that putatively encoded metal transporters (Table S2, Supporting Information) were amplified by using specific primers (Table S3, Supporting Information) and cloned into the pYES2 vector. The recombinant plasmids were expressed in *Δycf1*. The empty pYES2 vector was used as a negative control.

The transformed *Δycf1* yeast cells were precultured in liquid synthetic defined medium lacking uracil with 2% glucose and diluted to optical density at 600 nm (OD_600_) values of 1, 0.1, 0.01, 0.001, and 0.0001. The diluted yeast cells were cultured on solid synthetic galactose medium without uracil (SG‐U) supplemented with 2% galactose containing either 0 µmol L^−1^ CdCl_2_, 30 µmol L^−1^ CdCl_2_, 30 µmol L^−1^ NA, or 30 µmol L^−1^ Cd‐NA for four days. The plates were photographed after incubation.

To detect the Cd concentrations in yeast cells, the transformed yeast cells (100 µL, OD_600_ = 1.0) were added to SG‐U liquid medium containing 30 µmol L^−1^ Cd‐NA and incubated for 72 h. The yeast cells were then washed once using 50 mmol L^−1^ CaCl_2_ and three times with sterilized water to remove Cd from the cell surfaces. After centrifugation (10 000× *g*, 1 min, room temperature), the yeast cells were harvested, dried at 70 °C for four days, and used to determine the Cd concentrations. The Cd concentrations were determined in *Δycf1* yeast cells using inductively coupled plasma mass spectrometry (7700X, Agilent Technologies, Santa Clara, USA), as described in a previous study.^[^ ^35^ ^]^

### qRT‐PCR

The transcriptional levels of genes were determined by qRT‐PCR as described previously with minor modifications.^[^ ^38^ ^]^ Briefly, total RNA was isolated from wood samples exposed to 50 µmol L^−1^ CdCl_2_ for either 0 or 72 h using the CTAB method.^[^ ^39^ ^]^ Total RNA was purified using genomic DNA Eraser and reverse transcribed with a PrimeScript™ RT reagent Kit (RR047A, Takara, Dalian, China). Three or six biological replicates were conducted and each had three technical duplicates. *PcActin2/7* and *polyubiquitin* were used as reference genes.^[^ ^34^ ^]^ The gene expression levels were calculated according to a previously established method.^[^ ^40^ ^]^

### RNA in Situ Hybridization

The 8th internodes of four‐week‐old poplars were used for RNA in situ hybridization. To synthesize antisense and sense probes, PCR fragments of ca. 200 bp were amplified using gene‐specific primers (Table S3, Supporting Information). Hybridization and immunological detection were performed as described previously with minor modifications.^[^ ^41^ ^]^

### Plasmid Construction and Plant Transformation

To construct the GUS reporters, the promoter regions of *PcYSL3* and *PcWRKY1* of *P*. × *canescens* were separately amplified and cloned into the pKGWFS7 vector with the GUS reporter gene. For overexpression, the CDS regions of *PcYSL3* and *PcWRKY1* without the stop codon were separately cloned into the pCAMBIA2306 vector with the Flag tag via the *Xba*I site and driven by the CaMV35S promoter. To knock down *PcYSL3* and *PcWRKY1*, 253 bp of *PcYSL3* and 269 bp of *PcWRKY1* CDSs were separately cloned into the pFGC5941 vector via *Asc*I‐*Swa*I sites. Furthermore, the reverse complementary sequences of these fragments were inserted into the pFGC5941 vector between the *BamH*I‐*Xba*I sites, as described previously.^[^ ^42^ ^]^

The recombinant vectors were transformed into *P*. × *canescens* as described previously.^[^ ^6^ ^]^ Briefly, *P*. × *canescens* internodes were infected with *Agrobacterium tumefaciens* (Smith and Townsend 1907) Conn 1942 strain GV3101 containing recombinant constructs. Subsequently, the infected internodes were cultivated for three days in the dark. The infected internodes were transferred to new medium to produce shoots. Subsequently, the regenerated shoots were transferred to rooting medium. Successful transgenic production was verified by PCR using gene‐specific primers. Three overexpression and three knockdown lines of *PcYSL3* and *PcWRKY1* were selected for further analysis.

The transgenic and WT poplars were micropropagated. After rooting, poplars were cultivated by hydroponics in a greenhouse for two months, as described above. Three plants from each transgenic line and WT plants were treated with either 0 or 50 µmol L^−1^ CdCl_2_ for four weeks. The harvested stems were photographed to determine Cd‐induced damage. The 21st internodes were sampled for Cd histochemical staining. The wood and bark tissues were separated from the stems for further analysis.

### Histochemical Localization of GUS Expression

GUS staining was performed as described previously with minor modifications.^[^ ^43^ ^]^ Briefly, transgenic plants were fixed in 90% (v/v) acetone solution for 12 h at 4 °C and then washed three times with rinse buffer (50 mmol L^−1^ NaH_2_PO_4_, 50 mmol L^−1^ Na_2_HPO_4_ pH 7.2, 2 mmol L^−1^ K_3_Fe[CN]_6_, 2 mmol L^−1^ K_4_Fe[CN]_6_, and 0.1% Triton X‐100). GUS staining of plant tissues was visualized by incubating in rinse buffer containing 2 mmol L^−1^ 5‐bromo‐4‐chloro‐3‐indolyl‐β‐D‐glucuronide for 12 h in the dark at 37 °C. The plant tissues were decolorized using 75% (v/v) ethanol. GUS staining in stem cross sections was recorded under a microscope (SZX16, Olympus, Tokyo, Japan) connected to a camera.

### Western Blot

The western blot experiments were performed using wood samples from *PcYSL3_Pro_::GUS* and *PcWRKY1_Pro_::GUS* poplars as described previously with minor modifications.^[^ ^44^ ^]^ Briefly, one gram of wood from *PcYSL3_Pro_::GUS* or *PcWRKY1_Pro_::GUS* poplars exposed to Cd for either 0 or 72 h was ground in liquid nitrogen, and the powder was extracted for 10 min in 2× protein sample buffer. Subsequently, the proteins were centrifuged for 10 min at 16 000× *g*, separated on a 12% (w/v) polyacrylamide gel electrophoresis (PAGE) gel and transferred to the nylon membrane (FFN10, Beyotime, Shanghai, China). The nylon membranes were then blocked in Tris Buffered Saline Tween (TBST) buffer containing 5% (w/v) nonfat milk for 1 h and probed with anti‐GUS (P26299, Abmart, Shanghai, China) and anti‐Actin (M20009M, Abmart, Shanghai, China) in TBST. The secondary antibody, Goat Anti‐Rabbit IgG‐HRP (M21002L, Abmart, Shanghai, China), was diluted 1:3000 in TBST buffer. The membrane and secondary antibody were incubated at 37 °C with gentle shaking for 1–2 h. After three washes with TBST, signals were detected with BeyoECL Plus (P0018S, Beyotime, Shanghai, China). Imaging was performed using the ChemiDoc MP imaging system (Bio‐Rad Laboratories, California, USA).

### Determination of Subcellular Localizations of PcYSL3 and PcWRKY1

To determine the subcellular localizations of PcYSL3 and PcWRKY1, the CDS regions of *PcYSL3* and *PcWRKY1* were separately cloned into the pK7WGF vector with a green fluorescent protein (GFP) tag. The *GFP‐PcYSL3* recombinant vector and pMDC32–1A CAN2b‐mCherry (a plasma membrane marker) were co‐transformed into leaf epidermal cells of *N. benthamiana* by agroinfiltration.^[^ ^35^ ^]^ Similarly, *GPF*‐*PcWRKY1* was constructed and transferred into epidermal cells of tobacco leaves. After three days, leaves expressing *GFP‐PcWRKY1* were stained with 10 µg mL^−1^ 4′,6‐diamidino‐2‐phenylindole dye for 15 min, and then washed three times in de‐ionized water. Finally, the subcellular localizations of PcYSL3 and PcWRKY1 were observed by confocal laser scanning microscopy (LSM880, Carl Zeiss, Jena, Germany). Excitation/emission wavelengths of 488/510–530 nm, 587/600–620 nm, and 358/450–470 nm were used to detect green, red, and blue fluorescence, respectively.

To further examine whether PcYSL3 localized on the plasma membrane of ray parenchyma cells adjacent to the vessels, the subcellular localization of PcYSL3 was observed in the stems of *P*. × *canescens* by using immunoelectron microscopy, as described previously.^[^ ^23^ ^]^ The 8th internodes of *PcYSL3*‐overexpressing and WT poplars were used for immunoelectron microscopy assays. The stem cross sections (80 nm thick) were prepared and placed on copper grids, as described previously with minor modifications.^[^ ^23^ ^]^ The sections were incubated with mouse anti‐Flag antibody (ab125243, Abcam, Cambridge, UK) for 1 h at 25 °C and then overnight at 4 °C. Subsequently, the sections were incubated with goat anti‐mouse IgG antibody (10 nm gold, ab39619, Abcam, Cambridge, UK) for 1 h at 25 °C and stained with 6% (w/v) uranyl acetate. The sections were examined by transmission electron microscopy (HT7700, Hitachi, Tokyo, Japan).

### Histochemical and Fluorescent Staining of Cd

To characterize the localization of Cd in the stems, the sampled internodes described above were sectioned and stained using dithizone, as described in a previous study.^[^ ^45^ ^]^ An assay to determine Cd‐NA unloading from the vessels was conducted to confirm the transporter activity of PcYSL3 for Cd‐NA complexes in poplar wood. Briefly, the root systems were removed from transgenic and WT poplars aged four weeks to avoid Cd‐NA uptake via the root systems. The bases of these poplars were exposed to 250 µL of either 30 µmol L^−1^ NA or Cd‐NA solution for 1 h. Subsequently, the poplars were transferred to 250 µL of 100 µg mL^−1^ Leadmium™ Green AM solution for 1 h in the dark. Finally, the sections of the 8th internodes were observed by confocal laser microscopy.

### Determination of NA Concentrations

The NA concentrations were determined in wood samples from *PcYSL3*‐overexpressing, *PcYSL3*‐knockdown, and WT poplars as described previously.^[^ ^46^ ^]^

### Prediction of Upstream Transcription Factors Regulating PcYSL3 Expression

To identify upstream transcription factors that regulate *PcYSL3* expression, a three‐layer GRN was constructed as described previously with minor modifications.^[^ ^47^ ^]^ Briefly, the key putative genes encoding transporters, including *PcYSL3*, were selected and used to construct the third layer GRN. Upstream transcription factors were then predicted according to an algorithm proposed in a previous study.^[^ ^47^ ^]^ Subsequently, *cis*‐elements in the *PcYSL3* promoter region were identified using the JASPAR database (https://jaspar.genereg.net/, 20 September 2022). Transcription factors that could bind to the *cis*‐elements of *PcYSL3* were selected for further assays.

### Yeast One‐Hybrid Assay

Yeast one‐hybrid assays were performed according to a previously described method with minor modifications.^[^ ^30^ ^]^ Briefly, 1500 bp of the *PcYSL3* promoter sequence was cloned and inserted into the pHIS2 vector via the *EcoR*I site. The CDSs of putative transcription factors selected from the GRN mentioned above were cloned and ligated into the pGADT7 vector via the *EcoR*I site. The constructs were transformed into *S. cerevisiae* strain Y187. Transformed yeast cells were cultured in SD/‐Leu/‐Trp liquid medium and diluted to OD_600_ values of 1, 0.1, 0.01, 0.001, and 0.0001. The diluted yeast cells were plated on either SD/‐His/‐Leu/‐Trp solid medium supplemented with 80 mmol L^−1^ 3‐Amino‐1,2,4‐triazole or SD/‐Leu/‐Trp solid medium and incubated at 28 °C for four days. The plates were then photographed.

### EMSA

The EMSA assays were performed as described elsewhere with minor modifications.^[^ ^44^ ^]^ The CDS of *PcWRKY1* was cloned into the pET‐28a(+) vector with the His tag and transformed into *Escherichia coli* (Migula 1895) Castellani and Chalmers 1919 BL21. PcWRKY1‐His fusion protein was induced with 0.5 mmol L^−1^ of isopropyl‐b‐β‐thiogalactopyranoside for 12 h at 28 °C and purified using His‐tag Protein Purification Kit (P2226, Beyotime, Shanghai, China). Oligonucleotide probes containing the W‐box or mutant W‐box element were labeled with biotin at their 5′ ends by using EMSA Probe Biotin Labeling Kit (GS008, Beyotime, Shanghai, China).

EMSA was performed using the chemiluminescent EMSA Kit (GS009, Beyotime, Shanghai, China) according to the manufacturer's instructions. Unlabeled probes were employed as the competitors in competition analyses. The protein‐DNA mixtures were separated by electrophoresis on a 6% (w/v) PAGE gel and subsequently transferred to the nylon membrane. Signals were detected by chemiluminescence.

### MST Assay

The CDS of *PcWRKY1* was cloned and inserted into the pGEX4T1 vector with a GST tag and the empty pGEX4T1 vector acted as a negative control. The GST‐PcWRKY1 and GST proteins were expressed in *E. coli* BL21 cells and purified using glutathione agarose affinity chromatography (P2262, Beyotime, Shanghai, China).

MST assays were performed to detect the interactions between PcWRKY1 and each of P1, P2, P3, and P4 in the *PcYSL3* promoter according to a previously described method.^[^ ^48^ ^]^ The concentrations of Cy5‐labeled oligonucleotide probes were kept constant at 5 nmol L^−1^, whereas the concentrations of GST‐PcWRKY1 and GST were gradient diluted from 10 mmol L^−1^ to 0.61 nmol L^−1^. Diluted samples were incubated in MST buffer (50 mmol L^−1^ Tris, pH 7.6, 150 mmol L^−1^ NaCl, 10 mmol L^−1^ MgCl_2_, and 0.05% Tween‐20) for 5 min and loaded into MST‐standard glass capillaries. Measurements were performed using 1% LED power and 40% MST power with an MST system (Monolith NT.115, NanoTemper Technologies, Muenchen, Germany). Assays were repeated at least three times for each affinity measurement. Data analyses were performed using MO.Affinity software (version 2.2.4).

### ChIP and ChIP‐qPCR

ChIP assays were performed according to a previously described method with minor modifications.^[^ ^49^ ^]^ Briefly, the stem‐wood was harvested from WT and *P*. × *canescens* overexpressing *PcWRKY1*. The samples were cross‐linked in 1% formaldehyde under vacuum for 20 min, which was stopped with 0.125 mol L^−1^ glycine. Samples were ground into a fine powder in liquid nitrogen and nuclei were isolated. The chromatin was sheared into fragments of 200–1000 bp by sonication. Subsequently, the samples were divided into three groups, where one group was used as the input DNA sample, the second group was incubated with mouse anti‐Flag antibody (ab125243, Abcam, Cambridge, UK) for 12 h, and the third group was incubated with rabbit IgG (ab313801, Abcam, Cambridge, UK) for 12 h as a negative control. Finally, each DNA sample was analyzed by qPCR with *PcActin2/7* as a reference, and fold enrichment was calculated according to a previously described method.^[^ ^50^ ^]^ Three biological replicates were conducted and each had three technical duplicates.

### Dual‐Luciferase Reporter Assay

Dual‐luciferase reporter assays were performed as described previously with minor modifications.^[^ ^51^ ^]^ Briefly, 1445 bp of the *PcYSL3* promoter sequence was amplified and inserted into the pGreen II 0800‐LUC vector to generate a reporter construct. The CDS of *PcWRKY1* was cloned and inserted into the pGreen II 62 SK vector to construct an effector. The constructs were transferred into *A. tumefaciens* strain GV3101 with the pSoup plasmid. The *A. tumefaciens* effector and reporter were mixed 1:1 (v/v) and infiltrated into two distinct regions of the same *N. benthamiana* leaf. The luciferase activity was detected three days after infiltration. The expression levels of firefly and *Renilla* luciferases in *N. benthamiana* leaves were measured with a luminometer (GLOMAX® 20/20, Promega, Madison, USA). The ratio of firefly luciferase relative to *Renilla* luciferase (LUC/REN) was calculated to assess the final transcriptional activity. Three biological replicates were performed and each had three technical duplications.

### Statistical Analysis

Statistical analyses were performed using Statgraphics Centurion XVI.I (STN, St Louis, MO, USA). Analysis of variance (ANOVA) was conducted to detect significant differences in means between groups and to quantify the significance of independent over dependent variables. One‐way ANOVA was employed to compare the expression levels of genes including *PcCOPT5*, *PcZIP11*, *PcYSL3*, *PcWRKY1*, and *GUS*, the Cd concentrations in yeast cells and in wood tissues harvested at different time points, DNA fold enrichment in ChIP‐qPCR assays, and dual‐luciferase activities. Two‐way ANOVA was conducted to compare the Cd concentrations in different tissues treated with foliar Cd sprays, stem girdling, and transgenic and WT plants. Data normality was verified before conducting statistical analyses. Differences between means were considered significant when the *P*‐value was less than 0.05 according to the ANOVA F‐test. Posterior comparisons of means were performed using the least significant difference method.

## Conflict of Interest

The authors declare no conflict of interest.

## Author Contributions

X.C., Y.Z., and Y.C. contributed equally to this work. Z.L., S.D., and W.S. conceived the project and wrote the paper. Z.L., S.D., W.S., X.C., Y.Z., and Y.C. designed and performed the experiments. W.Y., L.Y., and P.S. contributed to sample preparation and statistical analysis. J.Z. and P.F. gave suggestions during the project.

## Supporting information



Supporting Information

Supporting Information

## Data Availability

The raw RNA‐sequencing data are deposited with links to BioProject PRJCA023618 at the National Genomics Data Center (https://ngdc.cncb.ac.cn).
