# Supplementary material for: PcWRKY1 Represses Transcription of Yellow Stripe‐Like 3 (PcYSL3) to Negatively Regulate Radial Cadmium Transport in Poplar Stems
Source: Adv Sci (Weinh). 2024 Nov 11;12(1):2405492. doi: 10.1002/advs.202405492 (PMC11714223; doi:10.1002/advs.202405492)
Supplement: Supplementary file 1 — Supporting Information [file ADVS-12-2405492-s002.pptx]

## Slide 1
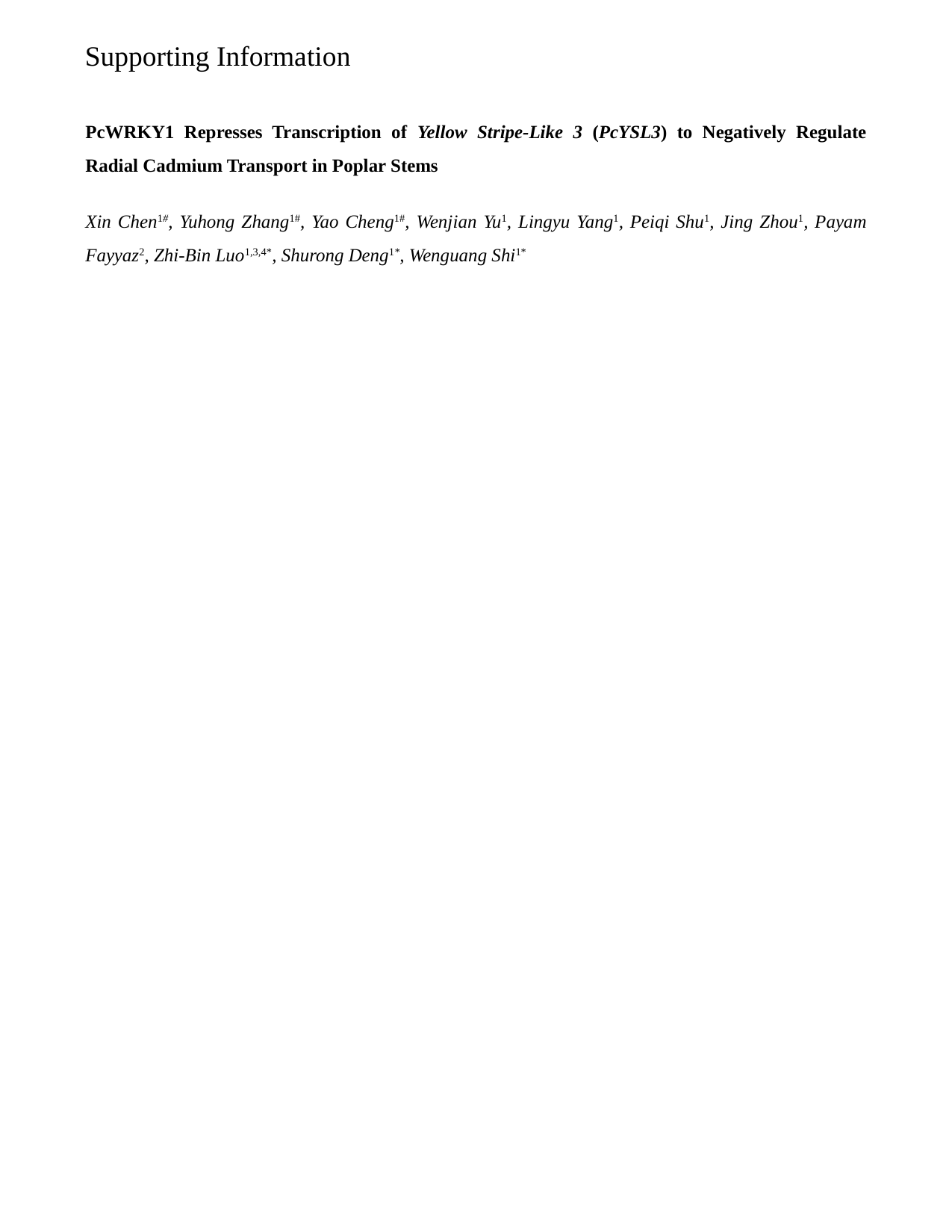

Supporting Information
PcWRKY1 Represses Transcription of Yellow Stripe-Like 3 (PcYSL3) to Negatively Regulate Radial Cadmium Transport in Poplar Stems
Xin Chen1#, Yuhong Zhang1#, Yao Cheng1#, Wenjian Yu1, Lingyu Yang1, Peiqi Shu1, Jing Zhou1, Payam Fayyaz2, Zhi-Bin Luo1,3,4*, Shurong Deng1*, Wenguang Shi1*

## Slide 2
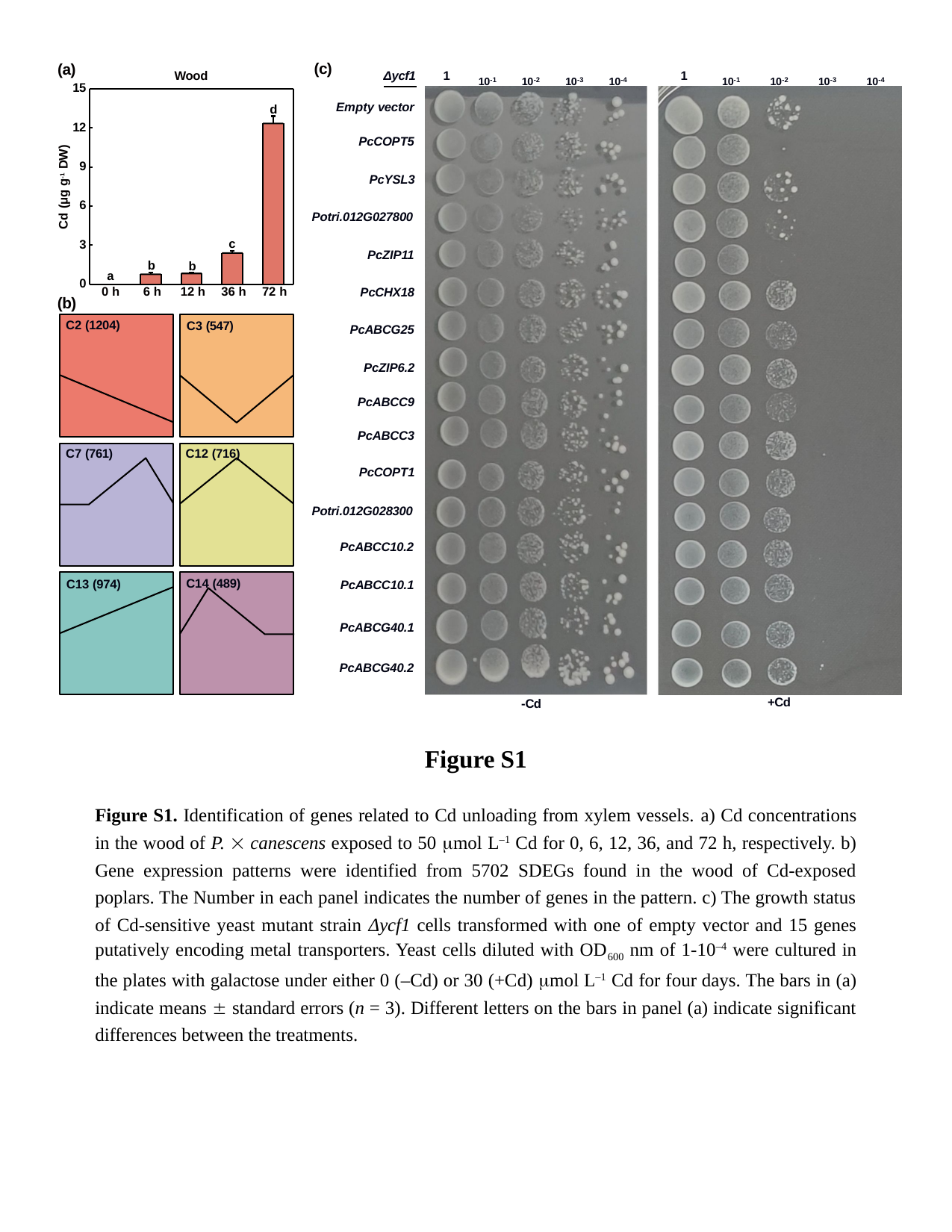

(a)
15
(c)
10-1
10-3
10-4
10-2
10-3
10-1
10-4
10-2
Wood
1
Δycf1	1
Empty vector
d
12
PcCOPT5
Cd (μg g-1 DW)
9
PcYSL3
6
Potri.012G027800
c
3
PcZIP11
b
b
a
0
0 h	6 h	12 h	36 h	72 h
C3 (547)
PcCHX18
(b)
C2 (1204)
PcABCG25
PcZIP6.2
PcABCC9 PcABCC3
C7 (761)
C12 (716)
PcCOPT1
Potri.012G028300 PcABCC10.2
C14 (489)
C13 (974)
PcABCC10.1
PcABCG40.1
PcABCG40.2
+Cd
-Cd
Figure S1
Figure S1. Identification of genes related to Cd unloading from xylem vessels. a) Cd concentrations in the wood of P. × canescens exposed to 50 μmol L–1 Cd for 0, 6, 12, 36, and 72 h, respectively. b) Gene expression patterns were identified from 5702 SDEGs found in the wood of Cd-exposed poplars. The Number in each panel indicates the number of genes in the pattern. c) The growth status of Cd-sensitive yeast mutant strain Δycf1 cells transformed with one of empty vector and 15 genes putatively encoding metal transporters. Yeast cells diluted with OD600 nm of 1-10–4 were cultured in the plates with galactose under either 0 (–Cd) or 30 (+Cd) μmol L–1 Cd for four days. The bars in (a) indicate means ± standard errors (n = 3). Different letters on the bars in panel (a) indicate significant differences between the treatments.

## Slide 3
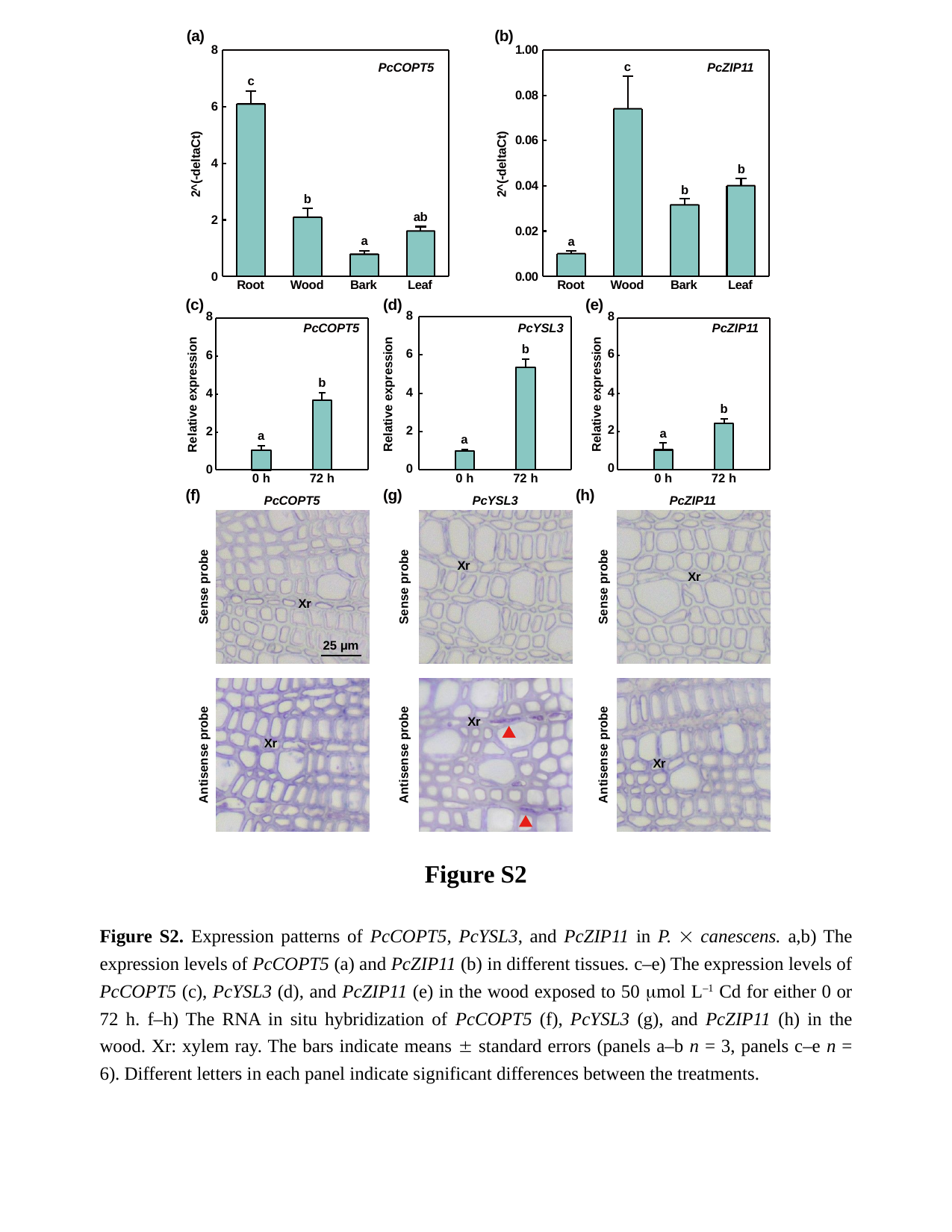

(a)
(b)
8
1.00
c
PcZIP11
PcCOPT5
c
0.08
6
2^(-deltaCt)
2^(-deltaCt)
0.06
4
b
0.04
b
b
ab
2
0.02
a
a
0.00
0
Root
(e)
Root
Wood
Bark
Leaf
Wood
Bark
Leaf
(c)
(d)
8
8
PcCOPT5
Relative expression
6
b
4
2
a
0
0 h
72 h
8
PcYSL3
b
PcZIP11
Relative expression
Relative expression
6
6
4
4
b
2
2
a
a
0
0
0 h
72 h
0 h
72 h
(f)
(g)
(h)
PcYSL3
PcZIP11
PcCOPT5
Sense probe
Sense probe
Sense probe
Xr
Xr
Xr
25 μm
Antisense probe
Antisense probe
Antisense probe
Xr
Xr
Xr
Figure S2
Figure S2. Expression patterns of PcCOPT5, PcYSL3, and PcZIP11 in P. × canescens. a,b) The expression levels of PcCOPT5 (a) and PcZIP11 (b) in different tissues. c–e) The expression levels of PcCOPT5 (c), PcYSL3 (d), and PcZIP11 (e) in the wood exposed to 50 μmol L–1 Cd for either 0 or 72 h. f–h) The RNA in situ hybridization of PcCOPT5 (f), PcYSL3 (g), and PcZIP11 (h) in the wood. Xr: xylem ray. The bars indicate means ± standard errors (panels a–b n = 3, panels c–e n = 6). Different letters in each panel indicate significant differences between the treatments.

## Slide 4
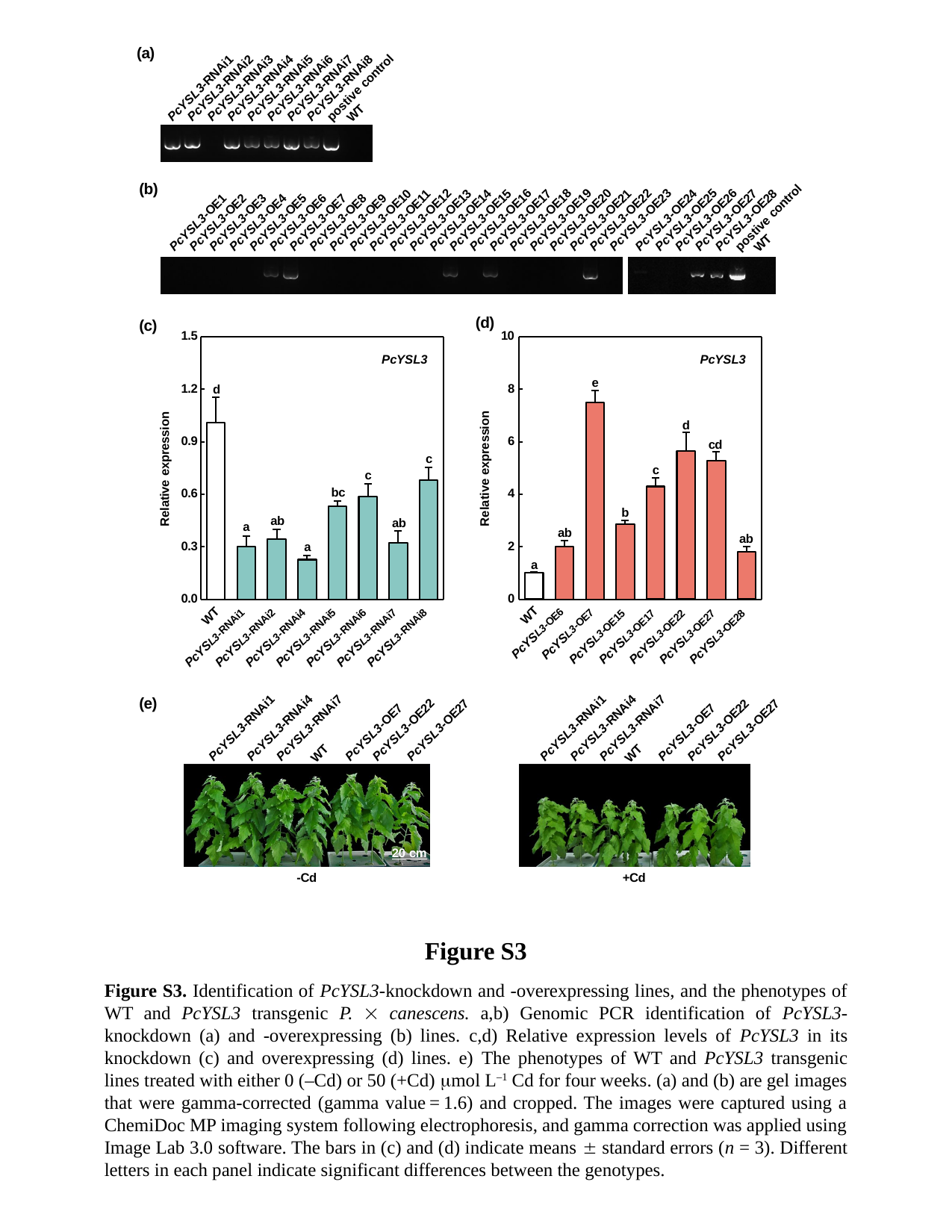

(a)
postive control
PcYSL3-RNAi8
PcYSL3-RNAi1
PcYSL3-RNAi2
PcYSL3-RNAi3
PcYSL3-RNAi4
PcYSL3-RNAi5
PcYSL3-RNAi6
PcYSL3-RNAi7
WT
(b)
postive control
PcYSL3-OE10
PcYSL3-OE12
PcYSL3-OE13
PcYSL3-OE14
PcYSL3-OE15
PcYSL3-OE16
PcYSL3-OE17
PcYSL3-OE18
PcYSL3-OE19
PcYSL3-OE20
PcYSL3-OE21
PcYSL3-OE22
PcYSL3-OE23
PcYSL3-OE24
PcYSL3-OE25
PcYSL3-OE26
PcYSL3-OE27
PcYSL3-OE28
PcYSL3-OE11
PcYSL3-OE1
PcYSL3-OE2
PcYSL3-OE3
PcYSL3-OE4
PcYSL3-OE5
PcYSL3-OE6
PcYSL3-OE7
PcYSL3-OE8
PcYSL3-OE9
WT
(c)
(d)
10
1.5
PcYSL3
PcYSL3
e
8
1.2
d
Relative expression
Relative expression
d
6
0.9
cd
c
c
c
bc
4
0.6
b
ab
ab
a
ab
ab
2
0.3
a
a
0
0.0
WT
WT
PcYSL3-OE6
PcYSL3-OE7
PcYSL3-OE15
PcYSL3-OE27
PcYSL3-OE17
PcYSL3-OE22
PcYSL3-OE28
PcYSL3-RNAi7
PcYSL3-RNAi2
PcYSL3-RNAi5
PcYSL3-RNAi6
PcYSL3-RNAi8
PcYSL3-RNAi4
PcYSL3-RNAi1
(e)
PcYSL3-RNAi1
PcYSL3-RNAi1
PcYSL3-RNAi4
PcYSL3-RNAi4
PcYSL3-RNAi7
PcYSL3-RNAi7
PcYSL3-OE22
PcYSL3-OE27
PcYSL3-OE22
PcYSL3-OE27
PcYSL3-OE7
PcYSL3-OE7
WT
WT
20 cm
-Cd
+Cd
Figure S3
Figure S3. Identification of PcYSL3-knockdown and -overexpressing lines, and the phenotypes of WT and PcYSL3 transgenic P. × canescens. a,b) Genomic PCR identification of PcYSL3-knockdown (a) and -overexpressing (b) lines. c,d) Relative expression levels of PcYSL3 in its knockdown (c) and overexpressing (d) lines. e) The phenotypes of WT and PcYSL3 transgenic lines treated with either 0 (–Cd) or 50 (+Cd) μmol L–1 Cd for four weeks. (a) and (b) are gel images that were gamma-corrected (gamma value = 1.6) and cropped. The images were captured using a ChemiDoc MP imaging system following electrophoresis, and gamma correction was applied using Image Lab 3.0 software. The bars in (c) and (d) indicate means ± standard errors (n = 3). Different letters in each panel indicate significant differences between the genotypes.

## Slide 5
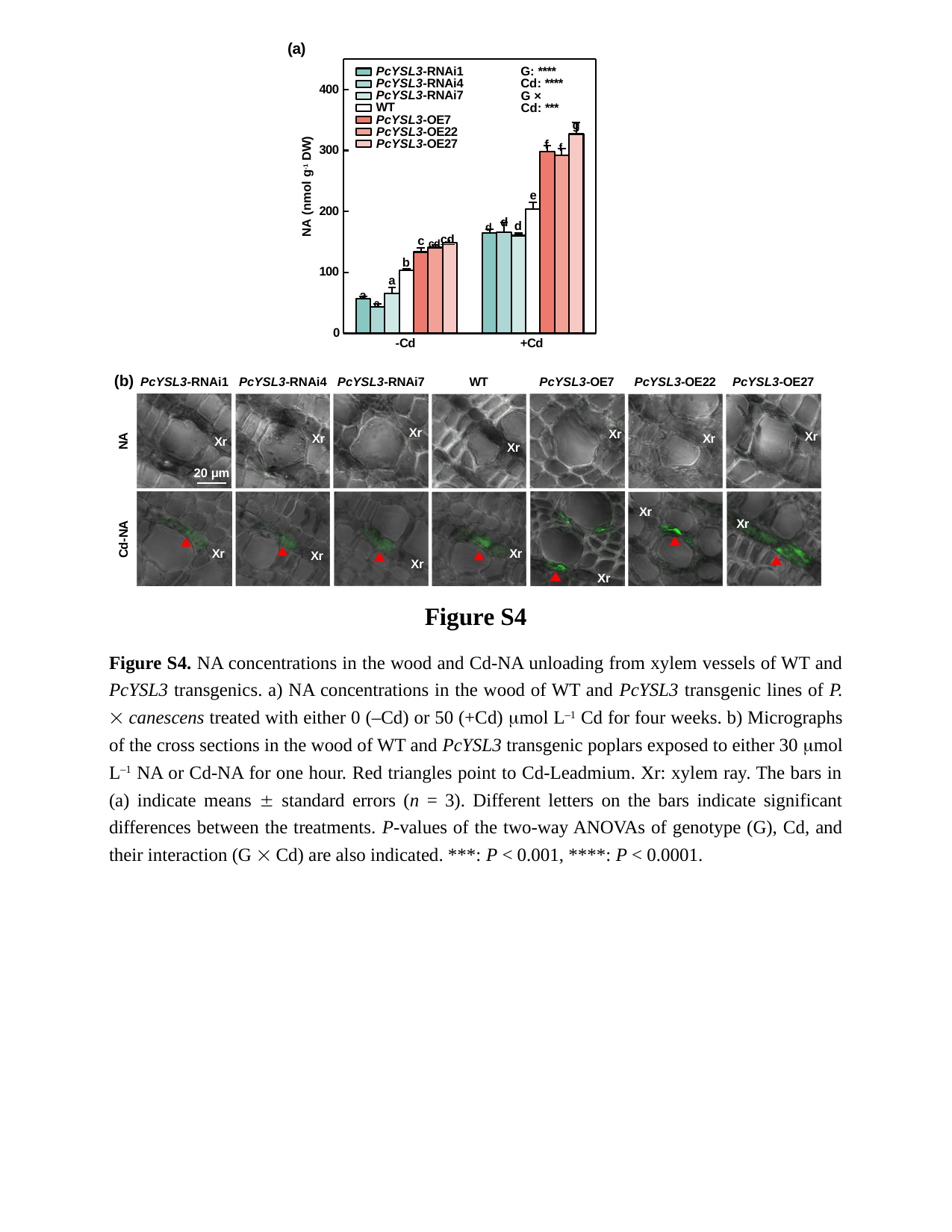

(a)
PcYSL3-RNAi1 PcYSL3-RNAi4 PcYSL3-RNAi7 WT
PcYSL3-OE7
G: **** Cd: ****
G × Cd: ***
g
400
PcYSL3-OE22
f f
PcYSL3-OE27
NA (nmol g-1 DW)
300
e
200
d d
d
cdcd
c
b
100
a
a a
0
-Cd
+Cd
(b) PcYSL3-RNAi1 PcYSL3-RNAi4 PcYSL3-RNAi7
WT
PcYSL3-OE7
PcYSL3-OE22
PcYSL3-OE27
Xr
Xr
Xr
Xr
Xr
NA
Xr
Xr
20 μm
Xr
Xr
Cd-NA
Xr
Xr
Xr
Xr
Xr
Figure S4
Figure S4. NA concentrations in the wood and Cd-NA unloading from xylem vessels of WT and PcYSL3 transgenics. a) NA concentrations in the wood of WT and PcYSL3 transgenic lines of P. × canescens treated with either 0 (–Cd) or 50 (+Cd) μmol L–1 Cd for four weeks. b) Micrographs of the cross sections in the wood of WT and PcYSL3 transgenic poplars exposed to either 30 μmol L–1 NA or Cd-NA for one hour. Red triangles point to Cd-Leadmium. Xr: xylem ray. The bars in (a) indicate means ± standard errors (n = 3). Different letters on the bars indicate significant differences between the treatments. P-values of the two-way ANOVAs of genotype (G), Cd, and their interaction (G × Cd) are also indicated. ***: P < 0.001, ****: P < 0.0001.

## Slide 6
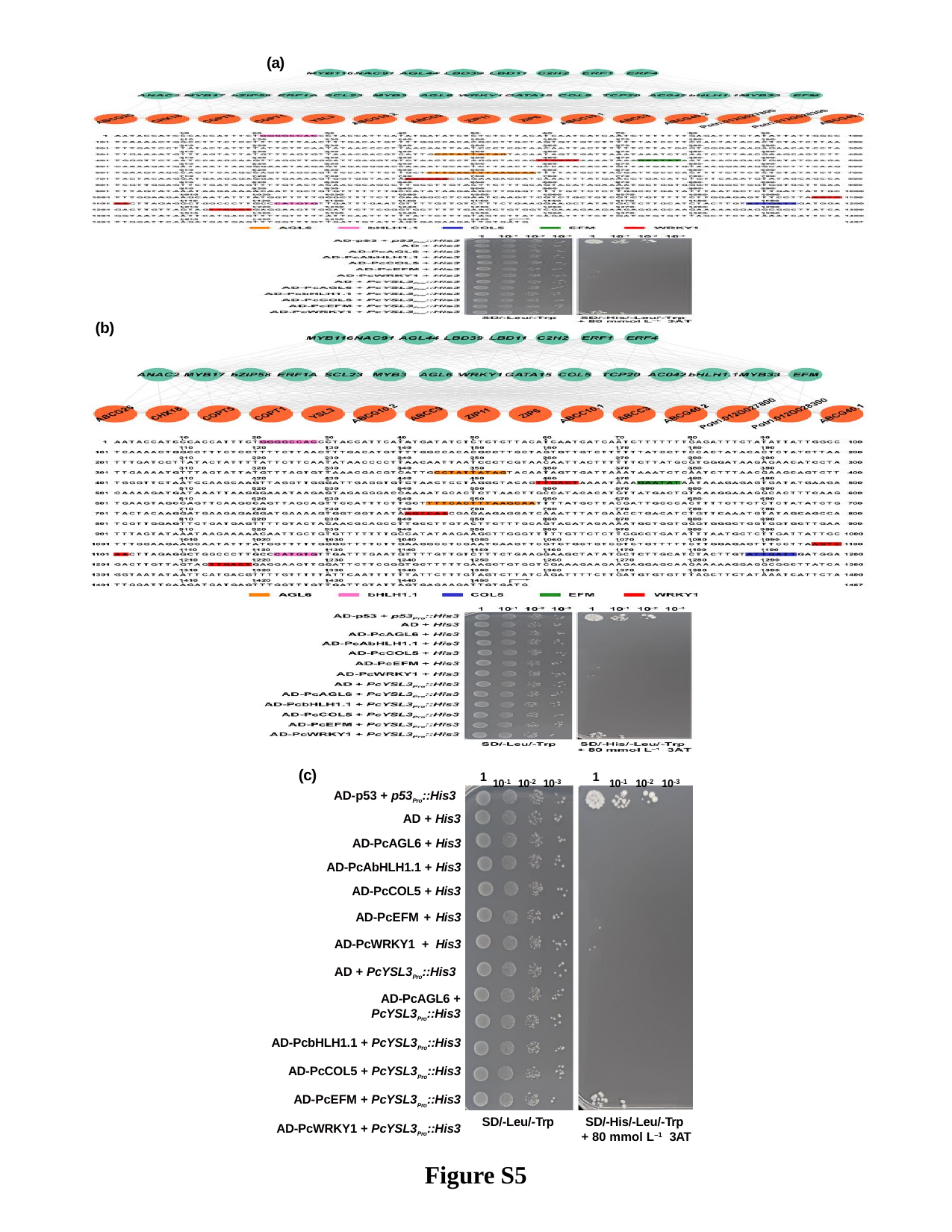

(a)
(b)
(c)
10-1 10-2 10-3	10-1 10-2 10-3
1	1
AD-p53 + p53Pro::His3
AD + His3
AD-PcAGL6 + His3 AD-PcAbHLH1.1 + His3 AD-PcCOL5 + His3
AD-PcEFM + His3 AD-PcWRKY1 + His3 AD + PcYSL3Pro::His3
AD-PcAGL6 + PcYSL3Pro::His3
AD-PcbHLH1.1 + PcYSL3Pro::His3 AD-PcCOL5 + PcYSL3Pro::His3 AD-PcEFM + PcYSL3Pro::His3 AD-PcWRKY1 + PcYSL3Pro::His3
SD/-Leu/-Trp
SD/-His/-Leu/-Trp
+ 80 mmol L–1 3AT
Figure S5

## Slide 7
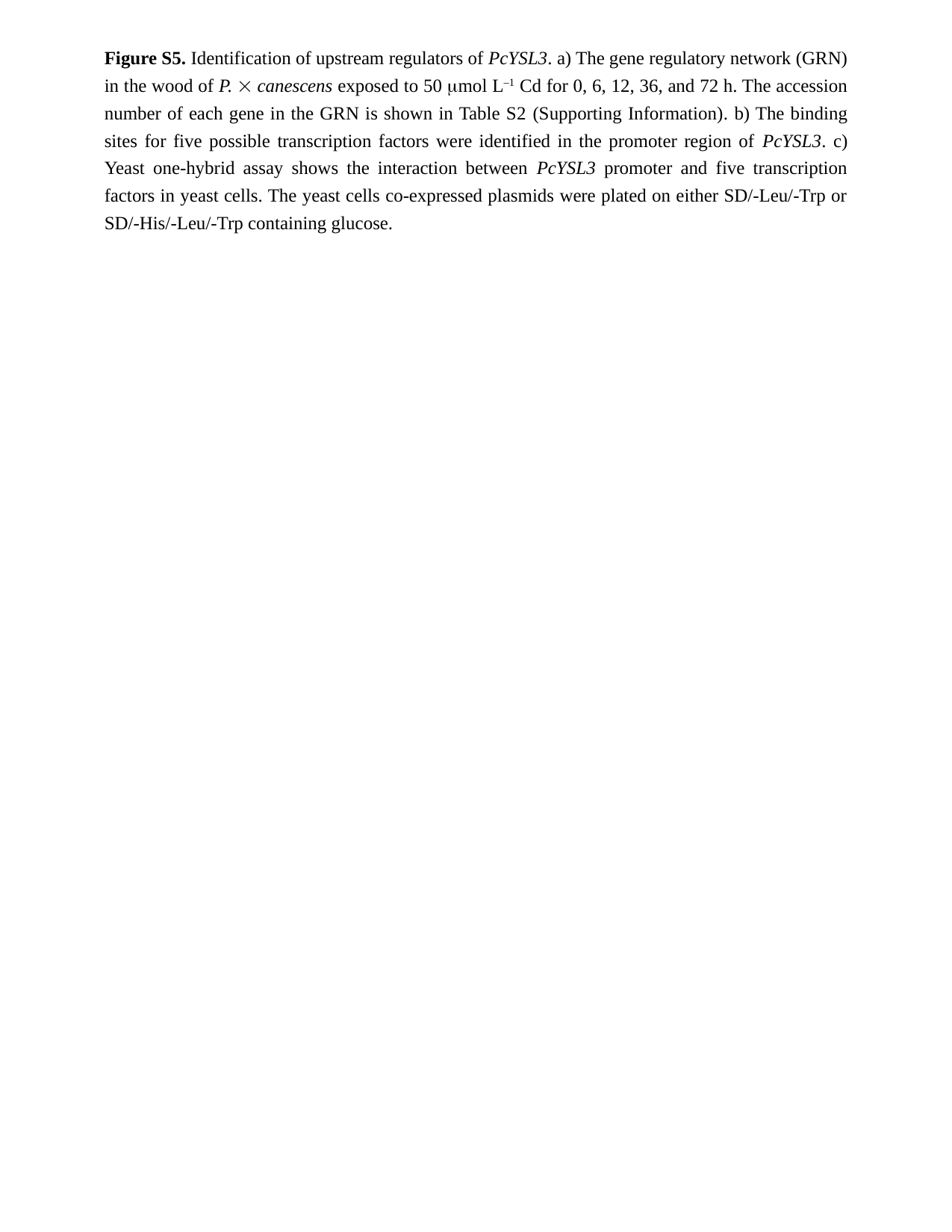

Figure S5. Identification of upstream regulators of PcYSL3. a) The gene regulatory network (GRN) in the wood of P. × canescens exposed to 50 μmol L–1 Cd for 0, 6, 12, 36, and 72 h. The accession number of each gene in the GRN is shown in Table S2 (Supporting Information). b) The binding sites for five possible transcription factors were identified in the promoter region of PcYSL3. c) Yeast one-hybrid assay shows the interaction between PcYSL3 promoter and five transcription factors in yeast cells. The yeast cells co-expressed plasmids were plated on either SD/-Leu/-Trp or SD/-His/-Leu/-Trp containing glucose.

## Slide 8
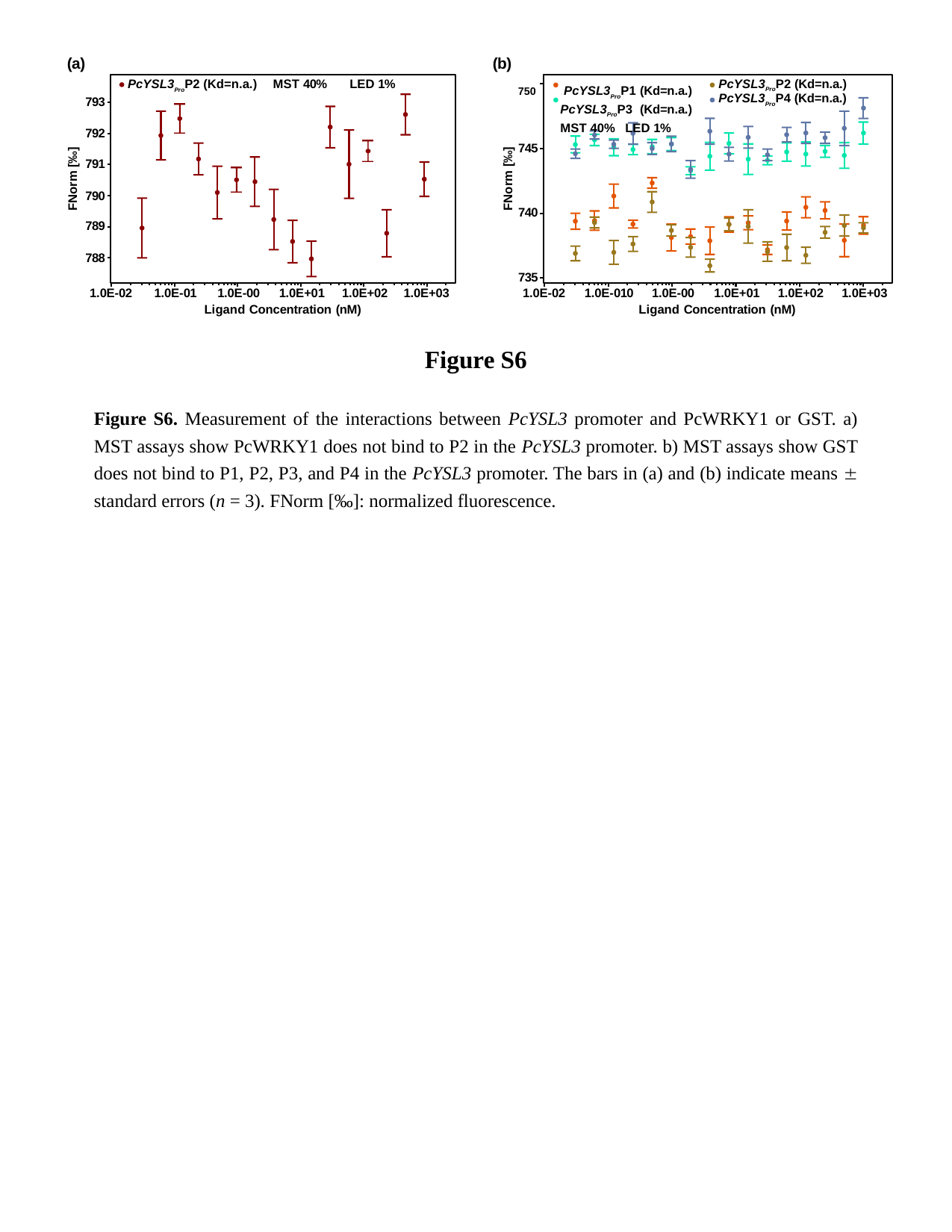

(a)
(b)
750 PcYSL3ProP1 (Kd=n.a.) PcYSL3ProP3 (Kd=n.a.) MST 40% LED 1%
PcYSL3ProP2 (Kd=n.a.) MST 40%	LED 1%
PcYSL3ProP2 (Kd=n.a.)
PcYSL3ProP4 (Kd=n.a.)
793
792
745
FNorm [‰]
FNorm [‰]
791
790
740
789
788
735
1.0E-02	1.0E-01	1.0E-00	1.0E+01	1.0E+02
Ligand Concentration (nM)
1.0E-02	1.0E-010	1.0E-00	1.0E+01	1.0E+02	1.0E+03
Ligand Concentration (nM)
1.0E+03
Figure S6
Figure S6. Measurement of the interactions between PcYSL3 promoter and PcWRKY1 or GST. a) MST assays show PcWRKY1 does not bind to P2 in the PcYSL3 promoter. b) MST assays show GST does not bind to P1, P2, P3, and P4 in the PcYSL3 promoter. The bars in (a) and (b) indicate means ± standard errors (n = 3). FNorm [‰]: normalized fluorescence.

## Slide 9
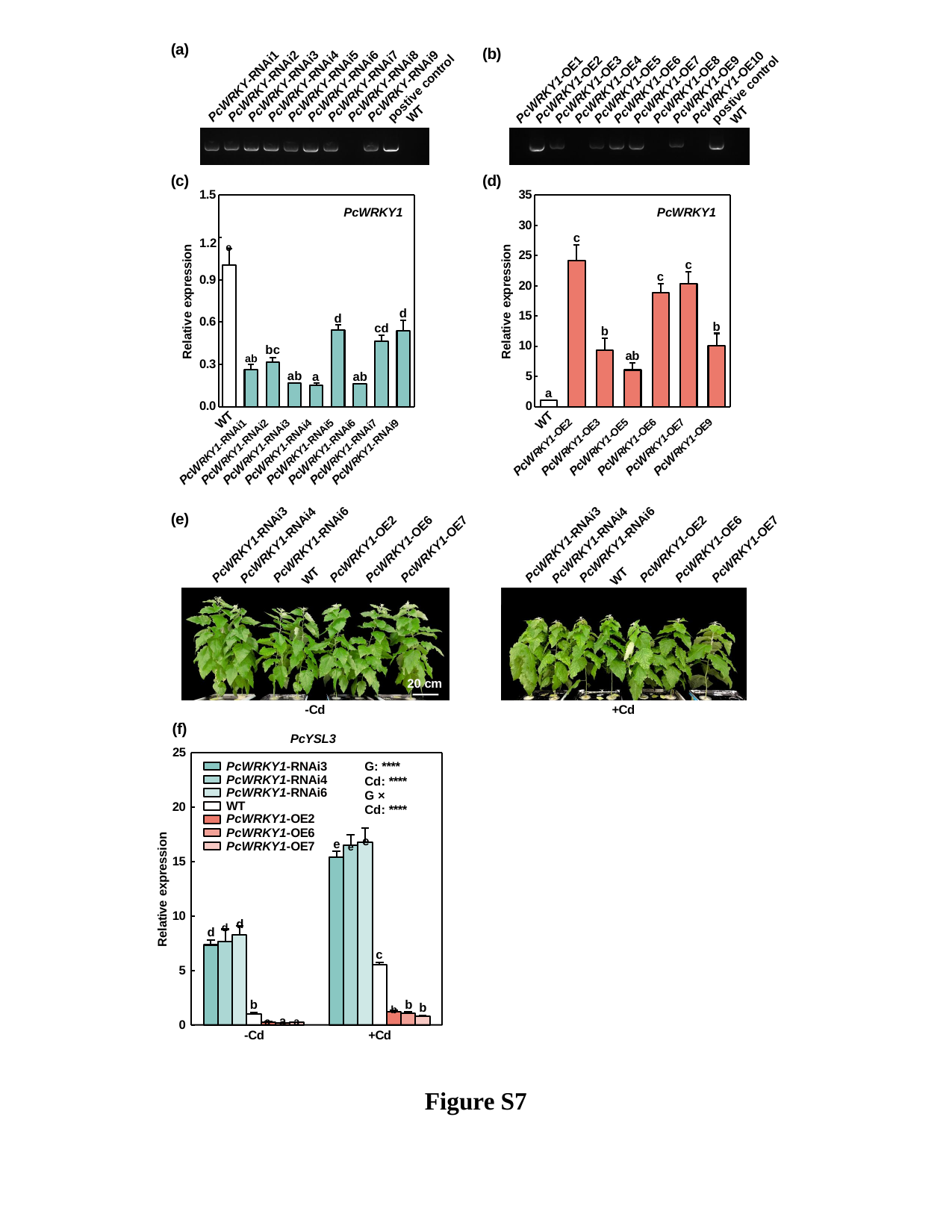

(a)
(b)
PcWRKY-RNAi1
PcWRKY-RNAi2
PcWRKY-RNAi3
PcWRKY-RNAi4
PcWRKY-RNAi5
PcWRKY-RNAi6
PcWRKY-RNAi7
PcWRKY-RNAi8
PcWRKY-RNAi9
PcWRKY1-OE10
postive control
postive control
PcWRKY1-OE1
PcWRKY1-OE2
PcWRKY1-OE3
PcWRKY1-OE4
PcWRKY1-OE5
PcWRKY1-OE6
PcWRKY1-OE7
PcWRKY1-OE8
PcWRKY1-OE9
WT
WT
(c)
(d)
35
1.5
PcWRKY1
PcWRKY1
30
1.2 e
c
Relative expression
Relative expression
25
c
c
0.9
20
d
15
d
0.6
b
cd
b
10
ab
bc
ab
0.3
5
ab
a
ab
a
0
0.0
WT
WT
PcWRKY1-OE3
PcWRKY1-OE2
PcWRKY1-OE5
PcWRKY1-OE6
PcWRKY1-OE7
PcWRKY1-OE9
PcWRKY1-RNAi1
PcWRKY1-RNAi2
PcWRKY1-RNAi3
PcWRKY1-RNAi4
PcWRKY1-RNAi5
PcWRKY1-RNAi6
PcWRKY1-RNAi7
PcWRKY1-RNAi9
(e)
PcWRKY1-RNAi6
PcWRKY1-RNAi3
PcWRKY1-RNAi6
PcWRKY1-RNAi3
PcWRKY1-RNAi4
PcWRKY1-RNAi4
PcWRKY1-OE2
PcWRKY1-OE2
PcWRKY1-OE6
PcWRKY1-OE7
PcWRKY1-OE6
PcWRKY1-OE7
WT
WT
20 cm
-Cd
+Cd
(f)
25
PcYSL3
PcWRKY1-RNAi3 PcWRKY1-RNAi4 PcWRKY1-RNAi6 WT
PcWRKY1-OE2
PcWRKY1-OE6
G: **** Cd: ****
G × Cd: ****
e e
20
Relative expression
e
PcWRKY1-OE7
15
10
d d
d
c
5
b
b
b
b
a a a
0
-Cd
+Cd
Figure S7

## Slide 10
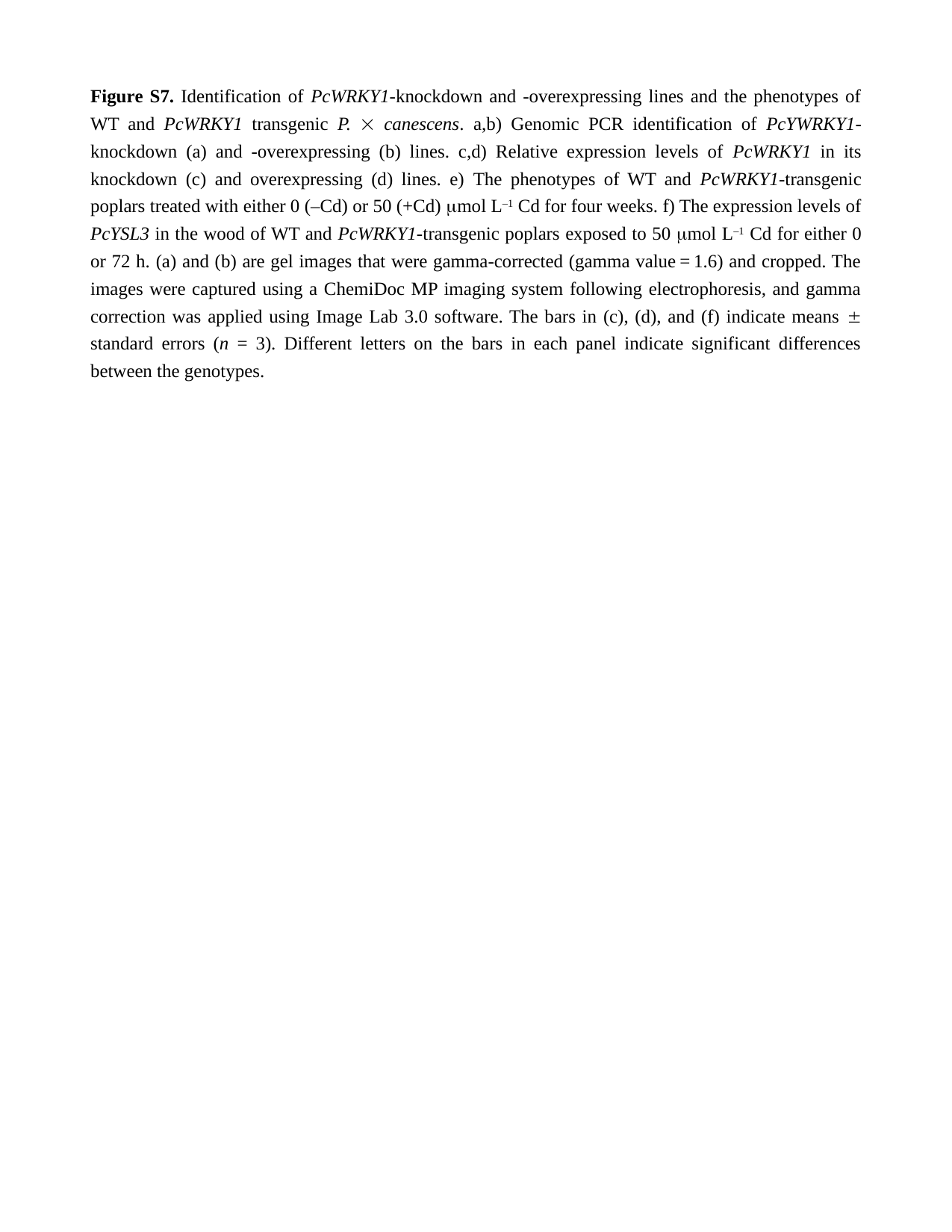

Figure S7. Identification of PcWRKY1-knockdown and -overexpressing lines and the phenotypes of WT and PcWRKY1 transgenic P. × canescens. a,b) Genomic PCR identification of PcYWRKY1-knockdown (a) and -overexpressing (b) lines. c,d) Relative expression levels of PcWRKY1 in its knockdown (c) and overexpressing (d) lines. e) The phenotypes of WT and PcWRKY1-transgenic poplars treated with either 0 (–Cd) or 50 (+Cd) μmol L–1 Cd for four weeks. f) The expression levels of PcYSL3 in the wood of WT and PcWRKY1-transgenic poplars exposed to 50 μmol L–1 Cd for either 0 or 72 h. (a) and (b) are gel images that were gamma-corrected (gamma value = 1.6) and cropped. The images were captured using a ChemiDoc MP imaging system following electrophoresis, and gamma correction was applied using Image Lab 3.0 software. The bars in (c), (d), and (f) indicate means ± standard errors (n = 3). Different letters on the bars in each panel indicate significant differences between the genotypes.

## Slide 11
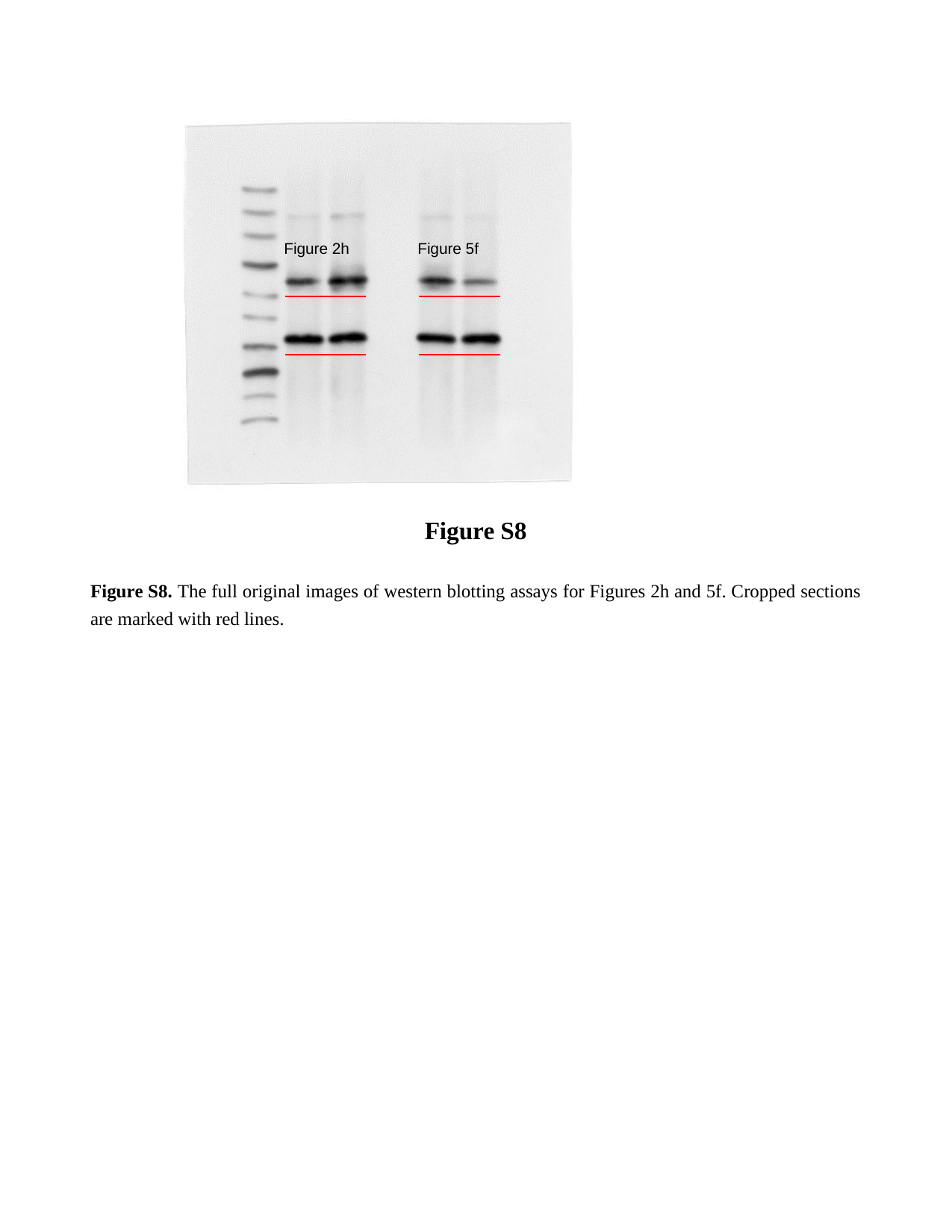

Figure 2h
Figure 5f
Figure S8
Figure S8. The full original images of western blotting assays for Figures 2h and 5f. Cropped sections are marked with red lines.

## Slide 12
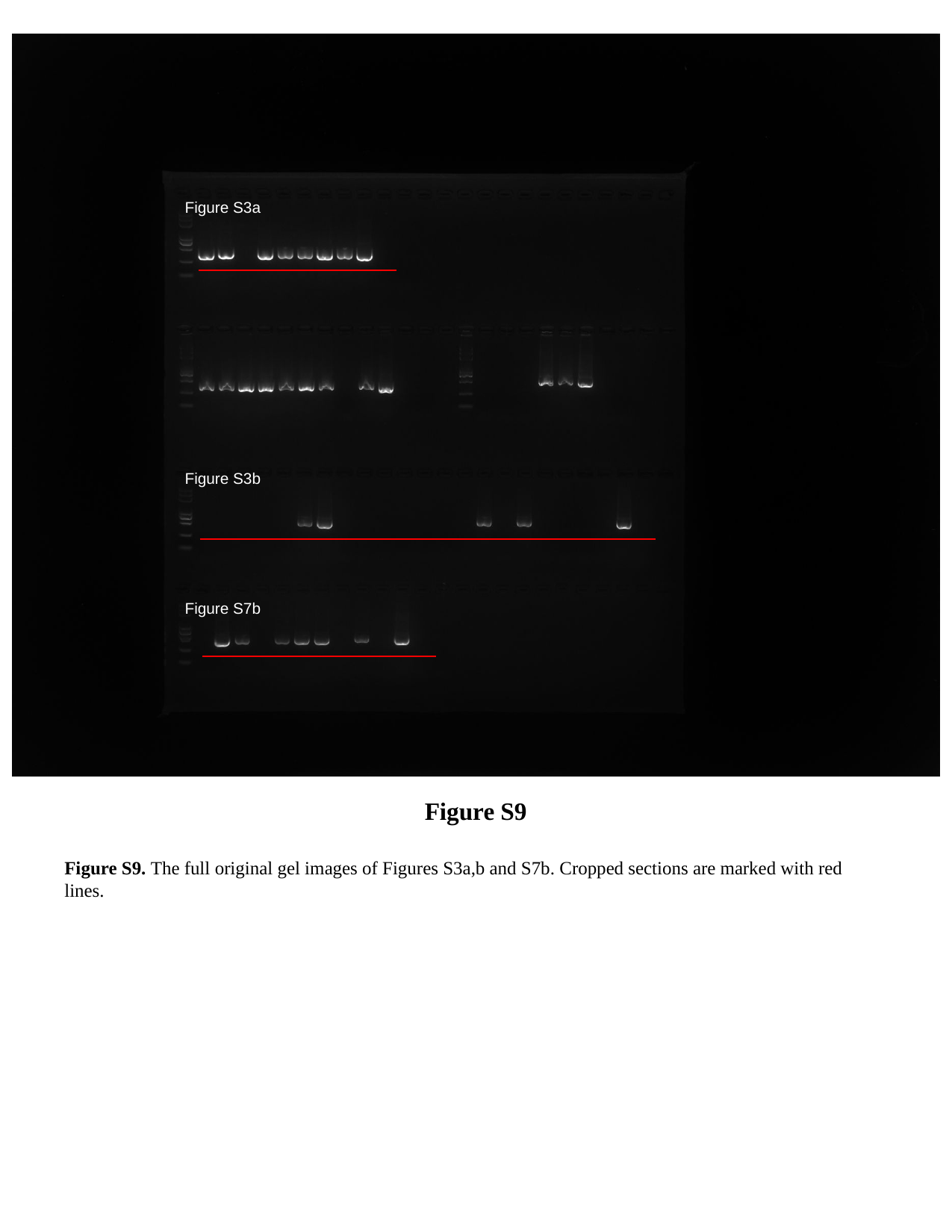

Figure S3a
Figure S3b
Figure S7b
Figure S9
Figure S9. The full original gel images of Figures S3a,b and S7b. Cropped sections are marked with red lines.

## Slide 13
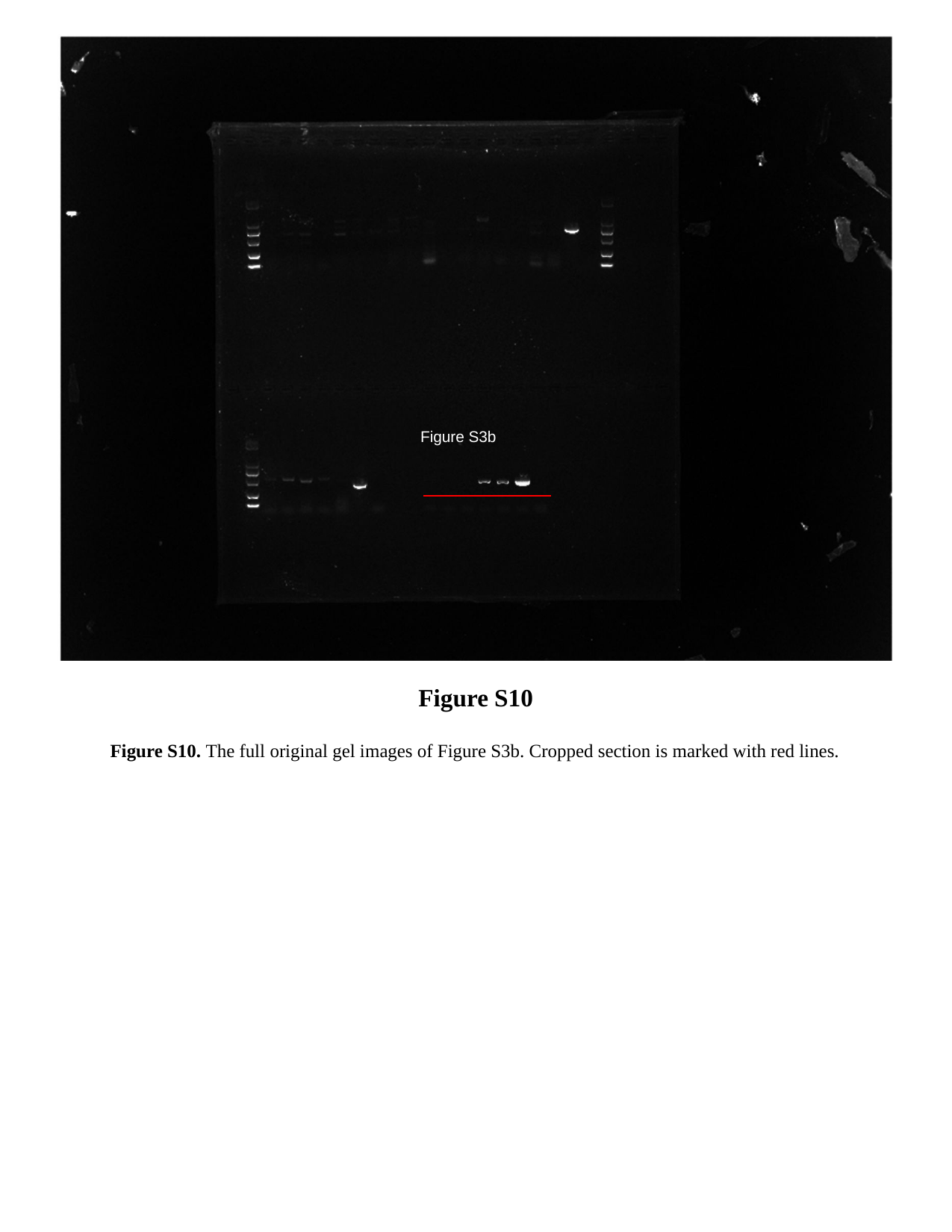

Figure S3b
Figure S10
Figure S10. The full original gel images of Figure S3b. Cropped section is marked with red lines.

## Slide 14
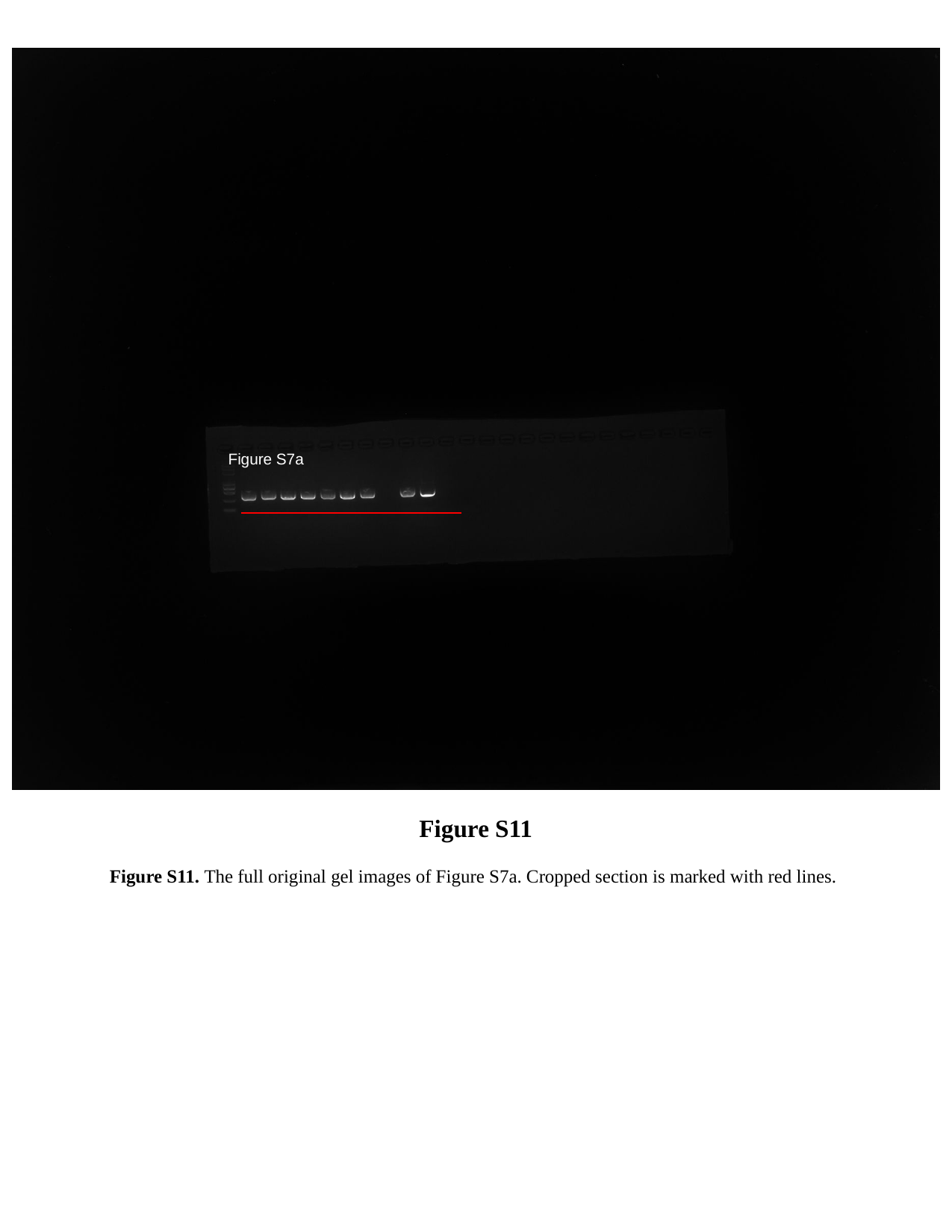

Figure S7a
Figure S11
Figure S11. The full original gel images of Figure S7a. Cropped section is marked with red lines.
